# A role of the CTCF binding site at enhancer Eα in the dynamic chromatin organization of the *Tcra–Tcrd* locus

**DOI:** 10.1093/nar/gkaa711

**Published:** 2020-08-27

**Authors:** Hao Zhao, Zhaoqiang Li, Yongchang Zhu, Shasha Bian, Yan Zhang, Litao Qin, Abani Kanta Naik, Jiangtu He, Zhenhai Zhang, Michael S Krangel, Bingtao Hao

**Affiliations:** Guangdong Provincial Key Laboratory of Tumor Immunotherapy, Cancer Research Institute, School of Basic Medical Sciences, Southern Medical University, Guangzhou, Guangdong 510515, P.R. China; Guangdong Provincial Key Laboratory of Tumor Immunotherapy, Cancer Research Institute, School of Basic Medical Sciences, Southern Medical University, Guangzhou, Guangdong 510515, P.R. China; Guangdong Provincial Key Laboratory of Tumor Immunotherapy, Cancer Research Institute, School of Basic Medical Sciences, Southern Medical University, Guangzhou, Guangdong 510515, P.R. China; Henan Medical Genetics Institute, People's Hospital of Zhengzhou University, Henan Provincial People's Hospital, Zhengzhou, Henan Province, China; Guangdong Provincial Key Laboratory of Tumor Immunotherapy, Cancer Research Institute, School of Basic Medical Sciences, Southern Medical University, Guangzhou, Guangdong 510515, P.R. China; Henan Medical Genetics Institute, People's Hospital of Zhengzhou University, Henan Provincial People's Hospital, Zhengzhou, Henan Province, China; Department of Immunology, Duke University Medical Center, Durham, NC, USA; State Key Laboratory of Organ Failure Research, National Clinical Research Center for Kidney Disease, Division of Nephrology, Nanfang Hospital, Southern Medical University, Guangzhou 510515, China; State Key Laboratory of Organ Failure Research, National Clinical Research Center for Kidney Disease, Division of Nephrology, Nanfang Hospital, Southern Medical University, Guangzhou 510515, China; Center for Biomedical Informatics, School of Basic Medical Sciences, Southern Medical University, Guangzhou 510515, China; Key Laboratory of Mental Health of the Ministry of Education, Guangdong-Hong Kong-Macao Greater Bay Area Center for Brain Science and Brain-Inspired Intelligence, Southern Medical University, Guangzhou 510515, China; Department of Immunology, Duke University Medical Center, Durham, NC, USA; Guangdong Provincial Key Laboratory of Tumor Immunotherapy, Cancer Research Institute, School of Basic Medical Sciences, Southern Medical University, Guangzhou, Guangdong 510515, P.R. China; Henan Medical Genetics Institute, People's Hospital of Zhengzhou University, Henan Provincial People's Hospital, Zhengzhou, Henan Province, China

## Abstract

The regulation of T cell receptor *Tcra* gene rearrangement has been extensively studied. The enhancer Eα plays an essential role in *Tcra* rearrangement by establishing a recombination centre in the Jα array and a chromatin hub for interactions between Vα and Jα genes. But the mechanism of the Eα and its downstream CTCF binding site (here named EACBE) in dynamic chromatin regulation is unknown. The Hi-C data showed that the EACBE is located at the sub-TAD boundary which separates the *Tcra–Tcrd* locus and the downstream region including the *Dad1* gene. The EACBE is required for long-distance regulation of the Eα on the proximal Vα genes, and its deletion impaired the *Tcra* rearrangement. We also noticed that the EACBE and Eα regulate the genes in the downstream sub-TAD *via* asymmetric chromatin extrusion. This study provides a new insight into the role of CTCF binding sites at TAD boundaries in gene regulation.

## INTRODUCTION

T lymphocytes recognize fragments of antigen with highly diverse T cell receptors (TCR) which are generated by V(D)J recombination during T lymphocyte development in thymus (1,2). V(D)J recombination of TCR genes occur in two stages of thymocyte development. First, the *Tcrg*, *Tcrd* and *Tcrb* genes undergo rearrangement in CD4^−^ CD8^−^ double negative (DN) thymocytes (3). TCRβ protein encoded by the successful rearranged *Tcrb* gene forms a pre-TCR heterodimer with pTα to drive cells into CD4^+^CD8^+^ double positive (DP) stage after several rounds of cell division. In DP cells, the *Tcra* gene undergoes several rounds of rearrangement on both alleles to generate TCRα, which replaces pTα to form αβTCRs that mediate positive and negative selection (1). Cells having passed these selection steps develop into CD4^+^ or CD8^+^ single positive cells and migrate from thymus.

The genes encoding Tcra and Tcrd shares a single genetic locus (*Tcra–Tcrd* locus), and the Tcrd gene is located between the Vα and Jα gene arrays. The *Tcrd* gene also shares some V gene segments with the *Tcra*. The regulation of *Tcra* rearrangement has been extensively studied (1,2). The previous studies showed that DP thymocytes establish a recombination centre at the Jα array with a high level of Rag protein binding, where Vα genes enter and undergo rearrangements with Jα genes (4,5). *cis*-regulatory elements such as enhancer Eα and the TEA promoter are involved in the recombination centre assembly (5). Chromatin organizers like cohesin and CTCF also play an essential role in *Tcra* rearrangement by mediating a chromatin loop between Eα and TEA (6,7). But it is unknown what drives the proximal Vα genes into the recombination centre from >200 kb upstream of the Jα array. It was reported recently that two CTCF binding sites INT1 and INT2 located at 10 kb upstream of Trdv4 suppress interactions between the proximal Vδ genes and Dδ genes in DN thymocytes (8).

It has been shown that Eα is necessary for establishing the recombination centre and for proximal Vα gene access to the centre (5,9). The deletion of a 1.1 kb region including the core Eα disrupted the chromatin hub which mediates the interactions between the Vα and Jα gene segments, blocked *Tcra* gene rearrangement, and arrested thymocyte development at DP stage (10). Notably, the 1.1 kb deletion not only removed the core Eα, but also removed the CTCF binding site (here named EACBE) at 500 bp downstream of the core Eα (11,12). It is unknown what specific roles the EACBE plays in recruiting proximal Vα genes into recombination centre and facilitating *Tcra* rearrangement.

It is well known that cohesin plays an essential role in chromatin organization and TADs (6,13–15). It was proposed that cohesin slides along chromatin to generate a DNA loop between two convergent CTCF binding sites (16,17). Vian *et al.* observed stripe-like architectures of interactions with anchors at the enhancers with CTCF binding sites in deep sequencing Hi-C heatmaps and proposed an asymmetric loop extrusion model in which cohesin is loaded at an enhancer with a CTCF binding site on one side and pulls DNA on the other side of the domain towards the enhancer (18). Recent studies showed that loop-extrusion-mediated Rag scanning played a critical role in the initiation of Igh rearrangement (19–21). Because Eα is located at the 3′ end of the *Tcra-Tcrd* locus with a CTCF binding site immediately downstream, asymmetric loop extrusion could play a role in locus architecture and *Tcra* rearrangement.

To explore its role in *Tcra* rearrangement and dynamic chromatin organization of the *Tcra–Tcrd* locus, we deleted the EACBE in the mouse genome. We found that the deletion impaired *Tcra* rearrangement. The Hi-C and 4C data showed that EACBE is essential for the sub-TADs boundary which separates the *Tcra–Tcrd* locus and the downstream region including *Dad1* gene. The deletion led the two sub-TADs to merge and reduced long-distance interactions between the proximal Vα genes and Jα array. We also noticed that the Eα and EACBE interact with the downstream region in the manner of a stripe-like architecture. Both the EACBE deletion and the EACBE plus core Eα deletion changed expression of the genes, especially the far downstream genes, in the downstream sub-TAD.

## MATERIALS AND METHODS

### Mice

Wild-type C57BL/6 mice were purchased from Guangdong Medical Animal Experimental Centre, Rag1^−/−^ and Rag2^−/−^ mice were kindly provided by Professor Wei Yang (Department of Pathology, Southern Medical University, Guangzhou, China). EACBE^−/−^ and LCR^−/−^ mice were generated from strain C57BL/6 by Beijing Vitalstar Biotechnology Co., Ltd. EACBE^−/−^ mice were bred with Rag1^−/−^ or Rag2^−/−^ mice to generate EACBE^−/−^ × Rag1^−/−^ or EACBE^−/−^ × Rag2^−/−^ mice. We crossed mice to get mice with C57BL/6 *Tcra/d* alleles. Mice were housed in a specific-pathogen-free facility managed by the Southern Medical University Division of Laboratory animal centre. Mice of both genders were included in all experiments and no differences on the basis of gender were noted. All mice were handled in accordance with protocols approved by the Southern Medical University Institutional Animal Care and Use Committee.

### Cell collection

Thymi, peripheral lymph nodes and spleens were generally collected from mice at 6 weeks of age and cells were filtered through a 40 μm nylon mesh and then pelleted. Red blood cells were removed by incubating in red cell lysis buffer (150 mM NH4Cl, 10 mM KHCO_3_, 0.1 mM EDTA, pH 7.4) for one minute. The reaction was quenched by adding MCAS buffer, pelleted by centrifugation, and resuspended in MACS buffer. To isolate DP thymocytes from Rag2^−/−^ mice, mice were injected i.p. with 150 μg of anti-CD3ϵ antibody (2C11; Biolegend) at 3 weeks of age and thymi were harvested ten days later. For T cell activation, isolated PLN and spleen T cell was cultured in anti-CD3/CD28 coated plates and FACS analyze was performed at 0 and 24 h, respectively.

### Flow cytometry and cell sorting

All antibodies were purchased from Biolegend unless stated otherwise. T cell development were detected by staining with antibodies to CD4(RM4–5), CD8α(53–6.7), CD44(IM7,BD), CD25(PC61), Thy1.2(53–2.1). γδT cell were detected by using anti-γδT(GL3), CD45RG(C363.16A), CD3(145–2C11) and lineage (Lin) CD4(GK1.5), CD8a(53–6.7), NK-1.1(PK136), CD49b(DX5), Ly-6G/Ly-6C(RB6–8C5), CD11b(M1/70), TER-119(TER-119), TCRβ(H57–597), CD19(1D3,eBioscience), B220 (RA3–6B2). T cell activation analysis were done by staining with antibodies to CD4(RM4–5), CD8α(53–6.7), CD25(PC61), Thy1.2(53–2.1). Data were acquired on a BD FacsCanto II flow cytometer in 8-color configuration and cell sorting was conducted using a Beckman Coulter Astrios or MoFlo.

### Preparation of *Tcra*, *Tcrd* and *Tcrb* repertoire sequencing libraries

10^7^thymocytes were lysed in Trizol (ThermoFisher) per manufacturer's specifications and either stored at −80°C or used immediately for RNA extraction. Total RNA was subjected to template-switch 5′ RACE as described (22,23), with modifications. Briefly, a mixture 10^7^ cell equivalents of RNA and 1 μM oligo(dT) primer in 8 μl nuclease-free water was heated to 65°C for 5 min and cooled down on ice for at least 5 min to snap-anneal the oligo(dT) primer. The reaction was then adjusted to 250 mM Tris–HCl (pH 8.3), 375 mM KCl, 15 mM MgCl_2_, 0.5 mM dNTPs and 5 mM dithiothreitol, before addition of 2 μl Superscript II (ThermoFisher), add 1 μl 25 uM 5′RACE adapter sequence (5′-GTCGCACGGTCCATCGCAGCAGTCACArGrGrG-3′) in a final volume of 20 μl. The reaction was incubated for 2 h at 50°C to synthesize cDNA and add RACE adapter by template switching. Reverse transcriptase was then inactivated by incubation at 85°C for 2 min.

PCR amplification of 5′RACE cDNA was performed as described (23), with modifications. All PCRs used Phanta polymerase in 2× Phanta mix (Vazyme, P511-02) in 50 μl total volume. PCR reactions contained 0.4μM antisense C region primer (Trac primer, 5′-TAAGGCGAACACAGCAGGTTCTGGGTTC-3′; Trdc primer, 5′-GAAAACAGATGGTTTGGCCG; Trbc primer, 5′-TAAGGCGAGGTGGAGTCACATTTCTCAG), and 0.4 μM sense RACE adapter primer (5′-TGAACCTTAAGCAGTGGTATCAACGCAGAG -3′), and 0.4 μM sense RACE adapter primer 2 (5′-ACGCTGACGCTGAGCCTACCTGAC-3′). PCR was performed using the program of one cycle of 3 min at 95°C, 30 cycles of 15 s at 95°C, 15 s at 60°C, 30 s at 72°C and one cycle of 5 min at 72°C. PCR products were run on 2% agarose gel, cut to get corresponding bands, extracted using QIAquick Gel Extraction kit (Qiagen) following manufacturer's specifications. After end repair, dA-tailing and linker ligation, barcodes and Illumina adapter sequences were then added by PCR amplification as described, with modifications (24). Sense primer sequence (5′-AATGATACGGCGACCACCGAGATCTACACTCTTTCCCTACACGACGCTCTTCCGATCT-3′). Antisense primer sequence (5′- CAAGCAGAAGACGGCATACGAGATNNNNNNGTGACTGGAGTTCAGACGTGTGCTCTTCCGATCT-3′) with Nextera × T N70X barcode. PCR products were subjected to the program of one cycle of 3 min at 95°C, 6–12 cycles of 15 s at 95°C, 15 s at 65°C and 30 s at 72°C, and one cycle of 5 min at 72°C. Several individual PCRs were run for each sample and products were purified by PCR Purification kit (Qiagen) then followed by size selection (0.6× plus 0.15× Ampure XP beads). PCR yields were quantified Qubit (ThermoFisher). The sequencing was done on an Illumina Hiseq X Ten platform using 150 bp pair-end reads at Beijing Novogene Technologies, Beijing, China. All primers and oligonucleotides were obtained from Thermo Fisher Scientific and Synbio Technologies, purified using standard desalting methods, and dissolved in nuclease-free water.

Reads were obtained following quality filtering and adaptor trimming using trim_glore (cutadapt version 0.4.4_dev) with parameter ‘-paired -q 20′. Individual reads were analyzed with MIXCR v2.1.10 to derive unique clonotypes based on CDR3 homology. Reads that comprise the clonotypes of interest (with matching VJ genes to Mus Musculus library antibody) were extracted for further analysis. The corresponding germline sequences were retrieved from IMGT, the international ImMunoGeneTics database. The total number of clonotypes was standardized to one million, and then calculated V–J clonotype ratio as V–J pairing usage.

### 4C-seq

3C sample preparation followed a previously described protocol with modification (25). In brief, 1 × 10 ^7^ cells were subjected to cross-linking by incubation for 10 min on ice in 10 ml of RPMI-1640 containing 10% FBS and 2% formaldehyde. The reaction was stopped by addition of glycine to 0.125 M and incubation for 5 min at room temperature. Cells were washed in PBS and lysed by incubation for 10 min on ice in 1 ml of cell lysis buffer (50 mM Tris, pH 7.5, 150 mM NaCl, 5 mM EDTA, 0.5% NP-40, 1% Triton X-100, one tablet protease inhibitor cocktail (Roche)). Nuclei were pelleted, washed once with PBS, and lysed by incubation for 1 h at 37°C in 0.5 ml of 1× CutSmart digestion buffer (New England Biolabs) containing 0.3% SDS. Triton-X100 was then added to a final concentration of 2% and incubation was continued for an additional 1 h at 37°C. Chromatin was then digested by addition of 400 U MboI for overnight incubation at 37°C. The MboI enzyme was inactivated by adding 80 μl 10% SDS and incubating at 37 °C for 30 min. Samples were transferred into 15 ml falcons and incubated with 4860 μl sterile water, 700 μl 10× ligation buffer and 750 μl 10% Triton X-100 for 1 h at 37 °C. 200 U of T4 Ligases (New England Biolabs) were added and incubated overnight at 16°C. De-crosslinking was performed by adding 30 μl of 10 mg ml^−1^ proteinase K for 4 h at 65°C. The DNA was precipated with 0.6× isopropanol and washed with 70% ethnal twice. DNA was dissolved in 150 μl of diluted TE.

3C samples were digested overnight at 37°C with 10 U of NlaIII. The digested libraries were purified by phenol–chloroform extraction, precipitated with 0.6× isopropanol, and rehydrated in 7 ml 30 mM Tris–HCl, pH 8.0, 10 mM MgCl_2_, 1 mM DTT and 0.1 mM ATP, after which 200 U of T4 DNA ligase (New England Biolabs) were added for overnight incubation at 16°C. 4C libraries were then purified by phenol-chloroform extraction, precipitated with 0.6× isopropanol, and rehydrated in 200 μl of 10 mM Tris–HCl, pH 8.0, 0.1 mM EDTA. Each library was then used for two rounds of inverse PCR from each of five viewpoints. First-round PCR was conducted with the following primers at 0.2 μM: Eα up-F (5′-TGGCGATGAAGTTGACTTTGATC-3′) and Eα up-R (5′-CAGGCAGAGACTCTTCGACG-3′), and Eα down-F (5′-TGCCCATCATCCAGGTTCAGATC-3′) Eα down -R (5′-CTGGGTTTGCTGCACCTCAGT-3′), and TEAp-F (5′-ACACTCTCTTTTCACAGCTGATC-3′) TEAp-R (5′-GCGTTCTGATTTCCTTCACTTTG-3′), and Trav17-F (5′-GACAAGGAACACAAACCCCGATC-3′) Trav17-R (5′-CCTGCTGTAATAATGTAGGAGGGC-3′) and Trav1-F (5′-GCTTCTGACAGAGCTCCAGATC-3′) Trav1-R (5′-GGAGCGTGGAAATGCTGTAGAT-3′), andINT-F (5′-ATTCAGCAGTGCTTTGTGTAGATC-3′) INT-R (5′-GGTTTCTGTGGTTGGAGTAGACT-3′). PCR conditions were as follows: 3 min at 95°C, followed by 30 cycles of 15 s at 95°C, 15 s at 55°C and 30 s at 72°C, with a final extension for 5 min at 72°C. PCR products were precipitated with 2× ethanol, dissolve the pellet in 50 μl nuclease-free water. Products were run on a 2% agarose gel, cut bands of 300–700 bp, extracted DNA by gel extraction kit (Qiagen) following manufacturer's specifications. The library preparation and sequencing followed the protocol described above in repertoire sequencing.

Reads were obtained following quality filtering and adaptor trimming using trim_glore (cutadapt version 0.4.4_dev) with parameter ‘-paired -q 20’. The first MboI enzyme fragment behind the viewpoint was extracted and mapped to mm10 by bowtie2 version 2.3.4.3 with parameter: –very-sensitive. Reads numbers were counted and normalized by the total mapped reads per sample after self-ligation remove, and then differential interactions were identified by 4C-ker with *k* = 30.

### *In situ* Hi-C

We generated *in situ* Hi-C libraries of DP thymocytes from WT and EACBE^−/−^ mice using the MboI restriction enzyme following the protocol described in (16) with small modifications. In brief, 10 × 10^6^ sorted DP thymocytes were crosslinked with formaldehyde, permeabilized, digested with MboI, filled-in with biotin-dCTP, ligated with T4 ligase and reverse crosslinked following the instruction of the *in situ* Hi-C method. DNA were resuspended in 130ul 10 mM Tris-HCl (pH 8.0) and sonicated to an average DNA fragment size of 300–500 bp (Q800R2 sonicator). 5 ug DNA dissolved in 130 μl 10 mM Tris–HCl (pH 8.0) was added into the pre-treated beads and incubated at RT for 15 min with rotation. The beads were then separated on a magnet and biotinylated DNA was bound to the streptavidin beads. After the ends of sheared DNA were repaired and the biotin from un-ligated ends was removed, adapters were added to the dA-tailed DNA fragments and PCR was performed with ten cycles using Illumina primers. Finally, DNA size selection was performed with 0.55–0.7× volume of VAHTS DNA Clean beads (Vazyme, N411-01-AA) to make the DNA length distribution between 300 and 500 bp. The library was quantified with Qubit and sequenced using an Illumina sequencing platform. Reads were obtained following quality filtering and adaptor trimming using trim_glore (cutadapt version 0.4.4_dev) with parameter ‘-paired -q 20’. Hi-C mapping, filtering, correction, and binning were performed with the HiC-Pro software v2.11.1. The reads were mapped to the mm10 mouse reference genome. TADs analysis was performed with the ‘Insulation Method’. A publicly available script (matrix2insulation.pl) was used to detect the TAD boundaries, with the following options: ‘–is 2000000 –ids 800000 –nt 0.3’. The script can be accessed through GitHub (https://github.com/dekkerlab/cworld-dekker).

### Crosslink ChIP

Crosslink ChIP was performed essentially as previously described (25). 1 × 10^7^ thymocytes were subjected to cross-linking by incubation for 10min on ice in 10 ml of 1× PBS containing 10% FBS and 1% formaldehyde (Pierce™ 16% Formaldehyde (w/v), ThermoFisher). The reaction was stopped by addition of glycine to 0.125 M and incubating for 5 min at room temperature. Cells were washed in PBS and lysed in by incubation for 5 min on ice in 1 ml of cell lysis buffer (50mM Tris pH 7.5, 150 mM NaCl,5mM EDTA, 0.5% NP-40, 1% TX-100,0.1 mM PMSF and 1× protease inhibitor cocktail (Roche)). After centrifugation, the nuclei were suspended in 40 μl of nuclei lysis buffer (50 mM Tris–HCl, pH 8.0, 10 mM EDTA, 1% SDS,0.1 mM PMSF and 1× protease inhibitor cocktail) and were broken by vigorous pipetting. The volume was then adjusted to 400 μl with nuclease-free water and the suspension was sonicated using a Model 550 Sonic Dismembrator (Fisher Scientific). Chromosomal DNA was reduced to an average size of 300–500 bp as determined by agarose gel analysis. The composition of the chromatin solution was then adjusted by addition of Tris–HCl pH 8.0, Triton X-100 and NaCl to 25 mM, 1.1% (v/v) and 170 mM, respectively. Chromatin was pre-cleaned by incubation for 30 min at 4°C with 5μg of IgG (R&D, AB-105-C) and 15μlprotein A/G magnetic beads (Pierce, 88802) twice. Pre-cleaned chromatin corresponding to 1 × 10^6^ thymocytes was incubated with 5 μg of specific antibody (anti-CTCF 07–729 from Millipore, anti-Rad21 Ab992 from Abcam, anti-RNA Pol II 05-623 from Millipore) or isotype-matched IgG for 16 h at 4°C, followed by addition of 15 μl protein A/G magnetic beads for an additional 4 h at 4°C. The supernatant was saved as the unbound fraction. Immunoprecipitates were washed by rocking for 5 min at 4°C twice each with 1 ml of the following buffers (containing protease inhibitors): (i) 0.01% SDS, 1.1% Triton X-100, 1.2 mM EDTA, 20 mM Tris–HCl pH 8.0, 170 mM NaCl; (ii) 0.1% SDS, 1% Triton X-100, 2 mM EDTA, 20 mM Tris–HCl pH 8.0, 500 mM NaCl; (iii) 1% NP-40, 1% deoxycholic acid, 100 mM Tris–HCl pH 8.0, 500 mM LiCl; and with 4) 10 mM Tris–HCl pH 8.0, 1 mM EDTA. DNA/Protein/Ab/beads complexes were resuspended by50 μl of 50 mM Tris (PH 8.0), 10 mM EDTA,1% SDS, 0.2mg/ml Proteinase K, rocking on Thermo mixer(ThermoFisher) for 1 h at 55°C, then 4 h at 65°C. After reversal of cross-linking and deproteination, place tube on magnet and collect supernatant purify by PCR purification kit. The standard curve quantification with gradient-concentrate genomic DNAs was used for quantifying ChIP products. qPCR primer are list in Supplementary Table S1.

### Native ChIP and ChIP-seq

Chromatin was prepared as previously described (21,25). Thymocytes (5–10.0 × 10^6^) were washed with cold PBS twice. Washed cells were then lysed in 200 μl of 80 mM NaCl, 10 mM Tris–HCl pH8.0, 10 mM sodium butyrate, 6 mM MgCl_2_, 1 mM CaCl_2_, 250 mM sucrose, 0.02% (vol/vol) NP40, 0.1 mM PMSF and 1× protease inhibitor cocktail for 5 min on ice. Centrifuge at 600 × g for 5 min at 4°C. Nuclei were pelleted and washed once with 10 mM NaCl, 10 mM Tris–HCl pH 8.0, 10 mM sodium butyrate, 3 mM MgCl_2_, 1 mM CaCl_2_ and 250 mM sucrose. Digestion was then performed to generate mainly mononucleosomes with a minor fraction of dinucleosomes, by incubation for 5 min at 37°C in 200 μl of the same buffer containing 6 U micrococcal nuclease (Worthington). The reaction was stopped by addition of 8μl of solution containing 0.2 M EDTA and 0.2 M EGTA. After centrifugation for 10 min at 18 000 × g, the supernatant was dilute into a final concentration of 16.7 mM Tris pH 8.0, 1.2 mM EDTA, 167 mM NaCl, 1.1% TritonX (vol/vol), 0.1 mM PMSF, and 1× protease inhibitor cocktail. Chromatin was then incubated overnight at 4°C with anti-acetylated H3 (Millipore 06-599), anti-trimethylated H3K4 (Millipore 04-745), anti-trimethylated H3K27 (Millipore, 07-449) or control rabbit IgG (R&D Systems, ab-105-c). protein A/G magnetic beads (Pierce,88802) was added for an additional four hours incubation, after which immunoprecipitates were washed vigorously and DNAs were purified. The standard curve quantification with gradient-concentrate genomic DNAs was used for qPCR. Immunoprecipitated and input DNAs were quantified by real time PCR using a StepOne™ Real-Time PCR System (Thermo Fisher,4376373) and a Hieff™ qPCR SYBR® Green Master Mix (YEASEN, China). For immunoprecipitation using anti-trimethylated H3K4 and anti-acetylation H3K27, analysis of *B2m* gene promoter was used as positive control and *MageA2* gene promoter was used as negative control to normalize ratios of bound/input in different samples. Primers sequences are provided in Supplementary Table S1. PCR conditions were as follows: 5 min at 95°C followed by 45 cycles of 30 s at 95°C, 1 min at 60°C. For Chip-seq, ChIP product through end repair, dA-tailing and linker ligation, barcodes and Illumina adapters were then added by PCR amplification. The libraries were purified with QiaQuick PCR purification reagents (Qiagen) and size selection by 0.7× and 0.2× Ampure XP beads (Beckman, A63880).

ChIP-seq libraries were pooled and sequenced via paired-end 150-bp reads on a HiSeq X TEN platform (Novogene). Reads were obtained following quality filtering and adaptor trimming using trim_glore (cutadapt version 0.4.4_dev) with parameter ‘-paired -q 20’. Mouse genome sequence (mm10) was retrieved from the UCSC (http://hgdownload.cse.ucsc.edu/goldenPath/mm10/bigZips/chromFa.tar.gz). PCR duplicate reads were removed by sambamba markdup with parameter ‘ –r –overflow-list-size 600000 ’. We used bowtie2 version 2.3.4.3 with the default parameters to align the reads to the mouse reference genome. Peak calling was done using Homer. The ChIP-seq read coverage was calculated using deepTools bamCoverage with RPKM normalization. The processing of the external data (Rad21, Nipbl, and CTCF ChIP-seq data from GSE48763 and GSE41743) was same as the new data.

### Germline transcription

RNA was extracted from unfractionated thymocytes using TRIzol (Invitrogen) according to the manufacturer's instructions. Contaminating genomic DNA was removed by incubation with 1U DNase I (New England Biolabs) for 10 min at 37°C. SuperScript reverse transcriptase (Invitrogen) and random hexamer primers were used to synthesize cDNA according to the manufacturer's instructions. Reverse transcription conditions were described previously (25). Real-time PCR was carried out using Relative Quantification strategy. Primers are listed in Supplementary Table S1.

### PCR and Southern blot analysis of rearrangements

Total thymocytes were lysed by incubation in 10 mM Tris–HCl pH 8.0, 150 mM NaCl, 10 mM EDTA, 0.4% (wt/vol) SDS and 0.1 mg/ml proteinase K overnight at 37°C. Genomic DNA was prepared by phenol/chloroform extraction and ethanol precipitation. PCR conditions were as follows: 3 min at 95°C; 30 cycles of 30 s at 95°C, 30 s at 60°C, 1 min at 72°C; 5 min at 72°C. After agarose gel electrophoresis and transfer to nylon membranes, PCR products were detected by hybridization with Biotin-labeled oligonucleotide probes. Primer and probe sequences are provided in Supplementary Table S1.

### Statistical methods

Data were analyzed by *t-*test, one-way ANOVA, or two-way ANOVA using Graphpad Prism software. *P* values of <0.05 were considered statistically significant. Sample sizes were estimated on the basis of initial experiments and measurements, rather than being predetermined on the basis of expected effect sizes. No data were excluded from analysis. There was no randomization of mice or ‘blinding’ of researchers to experimental groups.

## RESULTS

### The EACBE is located at the boundary of sub-TADs

**Figure 1.**
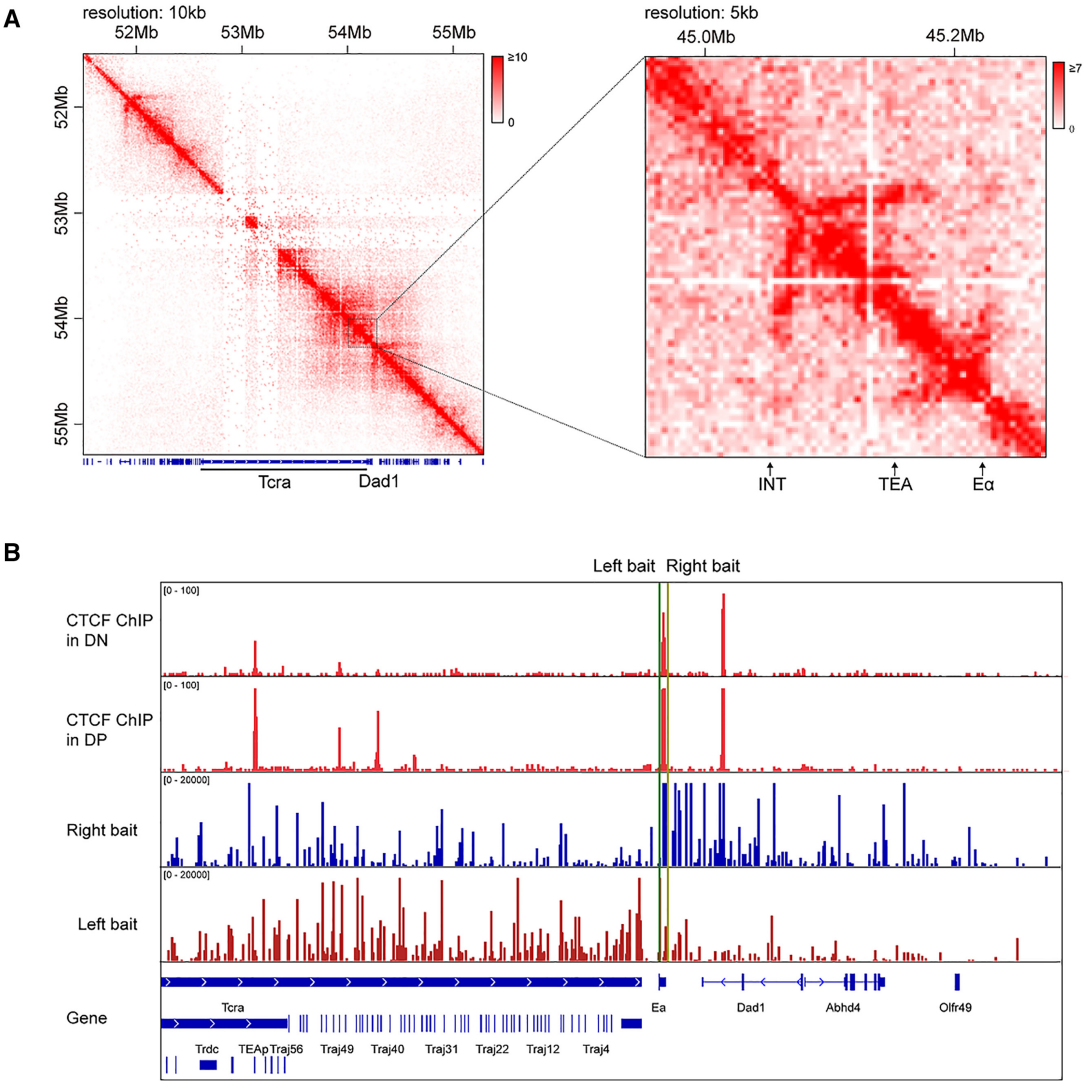
EACBE is located at the sub-TAD boundary. (**A**) Heatmap of 10 and 5 kb binned Hi-C data at the *Tcra–Tcrd* locus in DP thymocytes obtained from anti-CD3 injected Rag1^−/−^ mice. (**B**) CTCF ChIP-seq signal at the *Tcra–Tcrd* locus in DN and DP thymocytes of Rag2^−/–^ mice (up). 4C-seq profiles of interactions from the viewpoints of the left and the right of EACBE in DP thymocytes generated from anti-CD3 injected Rag2^−/−^ mice (down). The data are presented as reads per million mapped reads (RPM) within the *Tcra–Tcrd* locus. The green line and yellow line represent EACBE-left and EACBE-right viewpoint respectively. The 4C data are representative of two independent experiments.

To explore chromatin conformation of the *Tcra–Tcrd* locus in DP thymocytes, we performed Hi-C assays with DP thymocytes obtained from anti-CD3 injected Rag1^−/−^ mice. We observed that the enhancer Eα is located at the sub-TADs boundary which separates the *Tcra–Tcrd* locus and the downstream region including *Dad1* gene (Figure 1A). To clarify the function of the Eα CTCF binding element EACBE in the sub-TAD boundary, we designed two 4C primers located on the left and the right of the EACBE, respectively. The bait located on the left of EACBE displayed strong interactions with the Jα region in the leftward direction, while the right bait interacted with the *Dad1* gene (Figure 1B and Supplementary Figure S1). The results indicate an insulating function of the EACBE, which is consistent with reporter assay result in a previous report (26).

We observed that there was a stripe architecture anchored at the INT1–2 CBEs that extended far downstream in Hi-C heatmaps of several non-T cell types, including mESC (mouse embryonic stem cells), NPC (neural progenitor and cortical neurons), and neurons (Supplementary Figure S2a). This stripe is CTCF-dependent because it disappeared in the CTCF-degraded mESC cells, in which the CTCF was depleted with auxin-inducible degron system (Supplementary Figure S2b) (27). We observed that the stripe stops at the TEA promoter in DP thymocytes (Figure 1A), which suggests the loop extrusion from INT1–2 was blocked at the TEA promoter. We also observed a small stripe extending from Eα to TEA in DP thymocytes, suggesting that loop extrusion from INT1–2 and Eα converge on TEA to bring all three elements into proximity in DP thymocytes. Finally, we observed a stripe extending from Eα across downstream genes in DP thymocytes (Figure 1A), suggesting the possibility of a gene regulatory influence in the downstream direction.

### EACBE deficiency does not affect development of thymocytes

**Figure 2.**
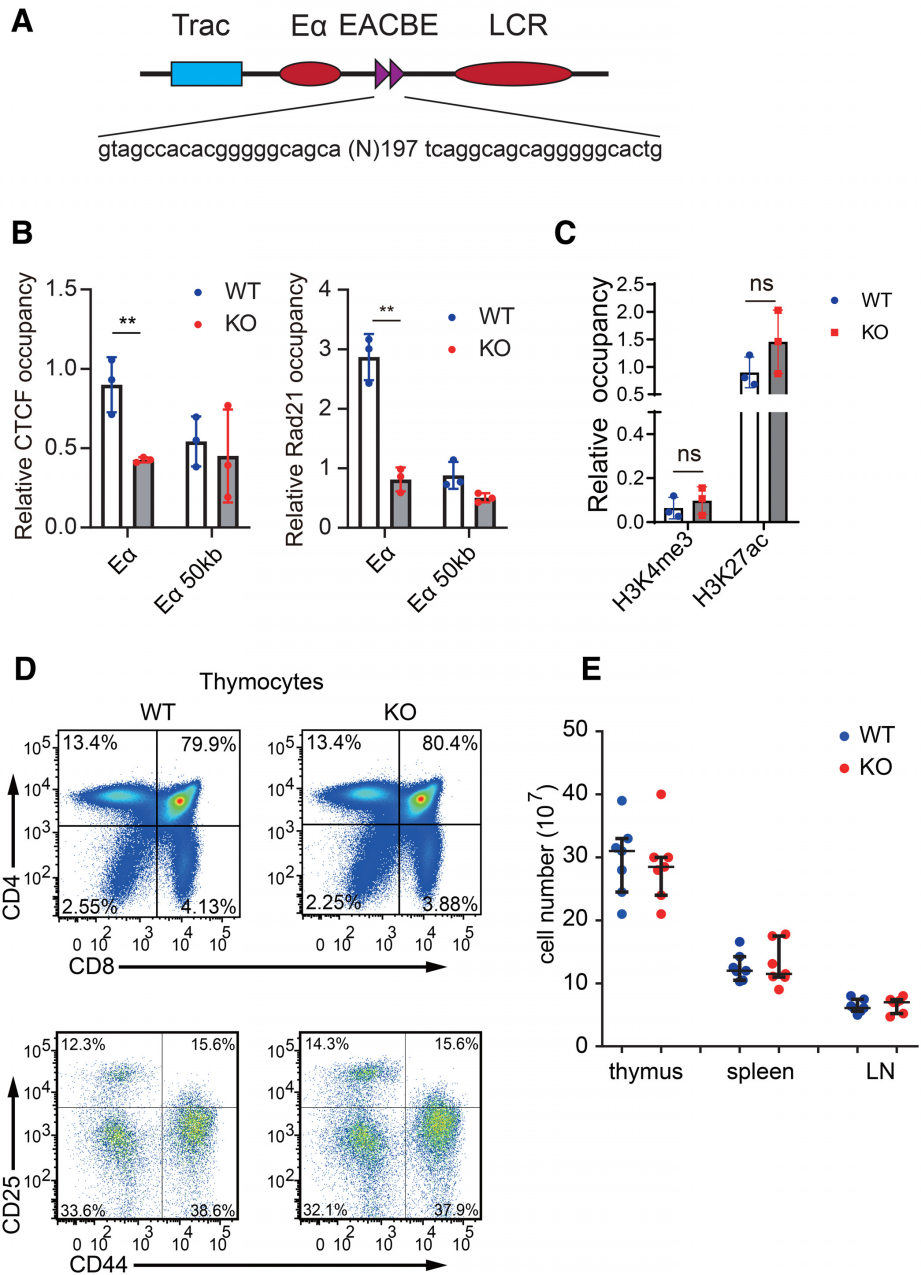
EACBE deletion doesn’t influence Eα activity and T cell development. (**A**) Schematic of the EACBE deletion. (**B**) CTCF and Cohesin occupancy on the Eα and negative control (50 kb downstream) were assessed by ChIP-qPCR. Values of bound/input were relative to the *Actb* gene promoter (normalized to 1). Data are representative of two independent experiments. The data represent mean ± SD of three PCR results of one experiment. **P <0.01 by two side Student's *t* test. (**C**) ChIP-qPCR of histone H3K4me3 and H3K27ac modification at Eα. Values of bound/input were relative to the *Actb* gene promoter (normalized to 1) in each sample. The data represent mean ± SD of three experiments. (**D**) Flow cytometric analysis of thymocyte subsets in 6 week-old wild type and EACBE^−/−^ mice. Data are representative of three independent experiments. (**E**) Cell numbers of thymocytes, spleen and lymph node (LN) in 6 week-old EACBE wild type and knockout mice (*n* = 6).

To test the role of the EACBE in *Tcra* rearrangement, we generated an EACBE-deleted allele in which a 197bp DNA fragment containing two CTCF binding motifs was deleted by using CRISPR-Cas9 system, leaving the core Eα intact (Figure 2A) (11). We compared the occupancy of CTCF and Rad21 at the Eα in thymocytes from WT and EACBE^−/−^ mice by using ChIP-qPCR. CTCF and Rad21 occupancy were reduced to the negative control level at Eα in EACBE-deficient thymocytes compared with that in WT cells (Figure 2B). To explore whether EACBE deletion changes Eα enhancer activity, we analyzed histone modifications H3K4me3 and H3K27ac at Eα in DP thymocytes (Figure 2C). EACBE deletion had no effect on either modification, suggesting that Eα was unperturbed by the deletion.

The EACBE^−/−^ mice have normal thymocyte development with comparable numbers of thymocytes as WT mice (Figure 2D, E and Supplementary Figure S3a-b). Splenic T cells and peripheral lymph node T cells are largely normal in 6-week-old EACBE^−/−^ mice as well (Figure 2E and Supplementary Figure S3c, d). Surface expression of TCRβ and CD3 are also similar on splenic T cells from EACBE^−/−^ and WT mice (Supplementary Figure S3e), indicating that EACBE isn’t involved in transcription of rearranged *Tcra* genes. To test whether EACBE^−/−^ mice display altered T cell activations, we sorted and stimulated naïve T cells from spleen and lymph node with anti-CD3/CD28 antibody. The result showed that EACBE deletion does not affect splenic T cell activation, while activation of lymph node T cells was moderately reduced (Supplementary Figure S4a–c). Taken together, the data showed that EACBE deficiency has no effect on the rearranged *Tcra* expression and thymocyte development, but a modest effect on T cell activation.

### EACBE deficient mice display impaired *Tcra* rearrangement

**Figure 3.**
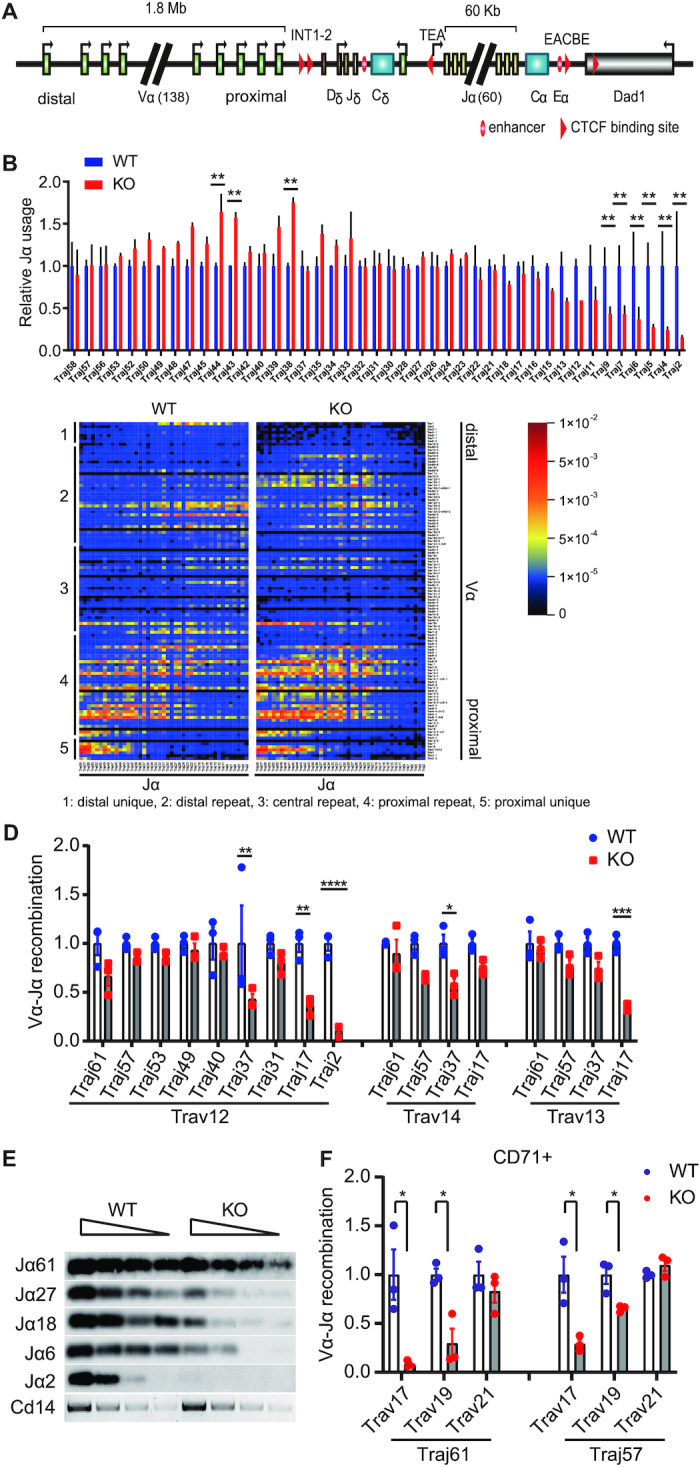
EACBE deletion impaired *Tcra* rearrangement. (**A**) Schematic of the Tcra-Tcrd locus. (**B**) Relative Jα usage and (**C**) Heatmap of Vα−Jα combination determined by high-throughput sequencing of *Tcra* transcripts amplified by 5′RACE of wild type and EACBE^−/−^ mice respectively. The relative Jα usage was calculated by dividing the number of the clonotypes containing the Jα gene by the total clonotype number. The data plotted as mean ± SD of two experiments. The signal in the heatmap is the number of the Vα−Jα clonotypes divided by the total clonotype number. Data are representative of two independent experiments. ** *P*<0.0005, by two side multiple Student's T test. (**D**) Jα usage was assessed by using qPCR with primers specific for Vα8 (Trav12), Vα2 (Trav14), or Vα10 (Trav13) families in conjunction with different Jα primers. Data are representative of three independent experiments. The data represent mean ± SD of three PCR results of one experiment. (**E**) Double strand breaks in two-fold serially diluted genomic DNA from wild type and EACBE^−/−^ thymocytes detected by ligation-mediated PCR and visualized by Southern blotting with Jα-specific probes. Data are representative of three independent experiments. (**F**) Vα–Jα combination quantified by using qPCR with Vα and Jα gene-specific primers in sorted CD71^+^ DP thymocytes. The qPCR values were determined by standard curve of thymus genomic DNAs. The data plotted as mean ± SD of three experiments, each with one mouse per genotype, with values for EACBE-deficient (KO) thymocytes normalized to those for wild type (WT) littermates. * *P*< 0.05, ** *P*< 0.01, **** *P*< 0.0001 by two side Student's *t* test.

To learn the pre-selected *Tcra* repertoire of EACBE deficient mice, we analyzed *Tcra* rearrangement by using a single primer pair targeting Cα and a common adapter added to the 5′ ends of cDNA during 5′ rapid amplification of cDNA ends (5′ RACE) (28). MiXCR immune repertoire analysis software was used for Jα usage and repertoire analysis. Only 43 of murine 60 Jα segments in the Jα array are functional due to their ability to undergo rearrangement and generate Tcra protein (Figure 3a). The Jα usage data clearly showed an abnormal pattern with reduced 3’ Jα usage in EACBE deficient thymocytes (Figure 3B). When we looked at Vα–Jα combinations of, we observed a clear bias of proximal Vα with 5′ Jα and distal Vα with 3′ Jα in WT thymocytes (Figure 3C), consistent with the previous report (28). We also observed combinations of central Vα with 5′ Jα. EACBE deficient mice displayed a similar overall pattern of rearrangements for the proximal and central Vα genes, but whereas rearrangements between the distal Vα and 3′ Jα genes were abundant in WT, they were reduced dramatically in EACBE deficient mice (Figure 3C). In addition, we noticed reduced usage of specific Vα genes in the proximal and central regions, includingTrav17, Trav8–2 and Trav14n-3 (Figure 3C). We also observed an abnormal increase in usage of some Vα genes in the distal repeat region (from Trav14d-1 to Trav7d-4) and reduced usage of the distal unique Vα gene Trav1 (Figure 3C). The results indicated that the EACBE is necessary for normal *Tcra* rearrangement and repertoire formation.

We also analyzed the repertoire of the *Tcrb* gene in WT and EACBE-deleted thymocytes. The results show that usage of Jβ and Vβ and combinations of Vβ-Jβ are exactly the same between WT and EACBE-deleted thymocytes (Supplementary Figure S5a–c), indicating a specific effect of EACBE on the *Tcra–Tcrd* locus rearrangement. It has been reported that *Tcrd* rearrangement shapes *Tcra* repertoire by deleting proximal V genes (8,28). We assessed the *Tcrd* repertoire of EACBE-deleted thymocytes by using RACE-seq. The usage of the proximal Vδ genes changed with reduced usage of the Trdv1 and Trdv2–2 and increased usage of Trdv5 (Supplementary Figure S5d). The results indicate that the EACBE also plays a role in *Tcrd* rearrangement, which may indirectly influence primary *Tcra* rearrangement.

We also tested *Tcra* V(D)J recombination by using qPCR to quantify Vα-to-Jα rearrangement in genomic DNA from WT and EACBE^−/−^ thymocytes. In EACBE^−/−^ thymocytes, the qPCR result revealed relatively normal rearrangement of Trav12, Trav14 and Trav13 to 5′ Jα gene segments, but impaired rearrangement of these Vα segments to the 3′ Jα segments (Figure 3D). This represents a deficiency in RAG-mediated cleavage at 3′ Jα gene segments rather than a deficiency in double-strand break repair, because similar reductions in signal end recombination intermediates at 3′ Jα gene segments were detected by ligation-mediated PCR (Figure 3E). Short life-span of thymocytes can lead to defective 3′ Jα usage (29). However, analysis of apoptosis and survival of thymocytes in culture revealed no such defect in EACBE-deleted thymocytes (Supplementary Figure S6a–c).

It was reported that CTCF deficiency impaired the primary rearrangement of the *Tcra* gene (7). We quantified early rearrangement of the two specific proximal Vα (Trav17, Trav19, and Trav21) and 5′Jα (Jα61 and Jα57) segments in CD71^+^ DP thymocytes, in which most of early Vα-Jα rearrangement occurs (Figure 3F). We observed a substantial reduction of the Trav17 rearrangement in EACBE deficient thymocytes, which is consistent with the RACE-seq result (Figure 3F). Trav19 rearrangement was also reduced 2–3-fold, while Trav21 kept same level (Figure 3F). The rearrangement of the proximal Vδ genes such as Trdv2–2 and Trdv1 deletes the INT1–2 region, which may promote the *Tcra* primary rearrangement. Reduced proximal Vδ gene rearrangement could partially explain the impaired primary *Tcra* rearrangement involving select proximal Vα genes.

### EACBE deletion changes accessibility of the proximal V genes but not the Jα array

**Figure 4.**
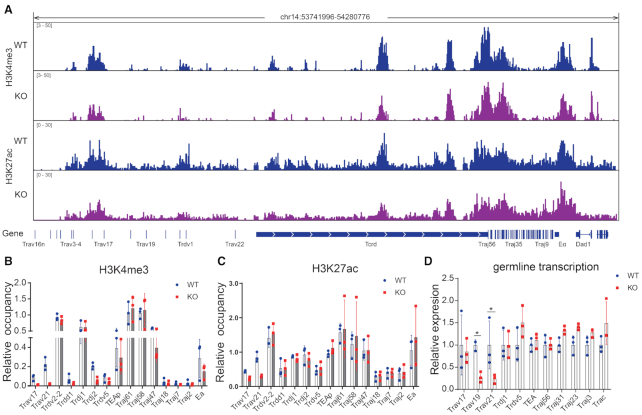
EACBE deletion reduced the accessibility of the proximal Vα genes. (**A**) H3K4me3 and H3K27ac ChIP-seq on the *Tcra*-*Tcrd* locus in Rag2^−/−^ (WT) and Rag2^−/−^ × EACBE^−/−^ (KO) DP thymocytes from anti-CD3 injected mice. Data are representative of two independent experiments. (**B**) and (**C**) Histone H3K4me3 and H3K27ac modification were analysed by ChIP qPCR. Values of bound/input were relative to the *Actb* gene promoter. (**D**) Relative transcription of the unrearranged Vα and Jα region was analysed using reverse-transcription qPCR. The expressions are normalized to the *Actb* gene and then to WT. The data represent the mean ± SD of three experiments. * *P* < 0.05 by two side multiple Student's *t* test.

The chromatin accessibility of the antigen receptor genes play an essential role in regulating V(D)J recombination (2). The EACBE may directly regulate initiation of the *Tcra* primary rearrangement in the *Tcrd*-unrearranged allele by regulating the histone modification and germline transcription of the *Tcra* gene. To explore this possibility, we detected histone H3K4 trimethylation and H3K27 acetylation of the *Tcra* locus by using ChIP-seq and ChIP-qPCR assay in DP thymocytes generated by injection of anti-CD3 antibody into Rag2 deficient mice (Figure 4A–C and Supplementary Figure S7a). We observed no significant difference in histone H3K4 trimethylation and H3K27 acetylation in the TEA and Jα regions of EACBE^−/−^ and WT mice (Figure 4A–C). We also checked the germline transcription and found that EACBE deletion didn’t reduce the TEA germline transcription, and even increased it slightly (Figure 4D). These observations are in contrast to those in Eα^−/−^ mice (in which Eα plus EACBE are deleted) (10,30), indicating that Eα regulates these properties of chromatin independent of EACBE.

Previous reports showed that the Eα-EACBE region contacts proximal Vα genes in CTCF-dependent manner and regulates their histone modifications, germline transcription, and rearrangement (7,31). H3K4 trimethylation at the two proximal Vα genes Trav17 and Trav21 reduced about four-fold in EACBE deficient DP thymocytes, although not statistically significant (Figure 4A and B). H3K27 acetylation level at the two Vα genes was also reduced slightly (Figure 4A and C). We also tested the germline transcription of the three proximal Vα genes and found that the Trav19 and Trav21 displayed a 2- to 3-fold reduction of germline transcription in EACBE deficient thymocytes (Figure 4D). The reduced accessibility of the proximal Vα gene region indicated a direct and specific contribution of the EACBE to the regulation of select Vα segments on *Tcrd*-unrearranged alleles.

### EACBE insulates the *Tcra–Tcrd* locus from downstream genes

**Figure 5.**
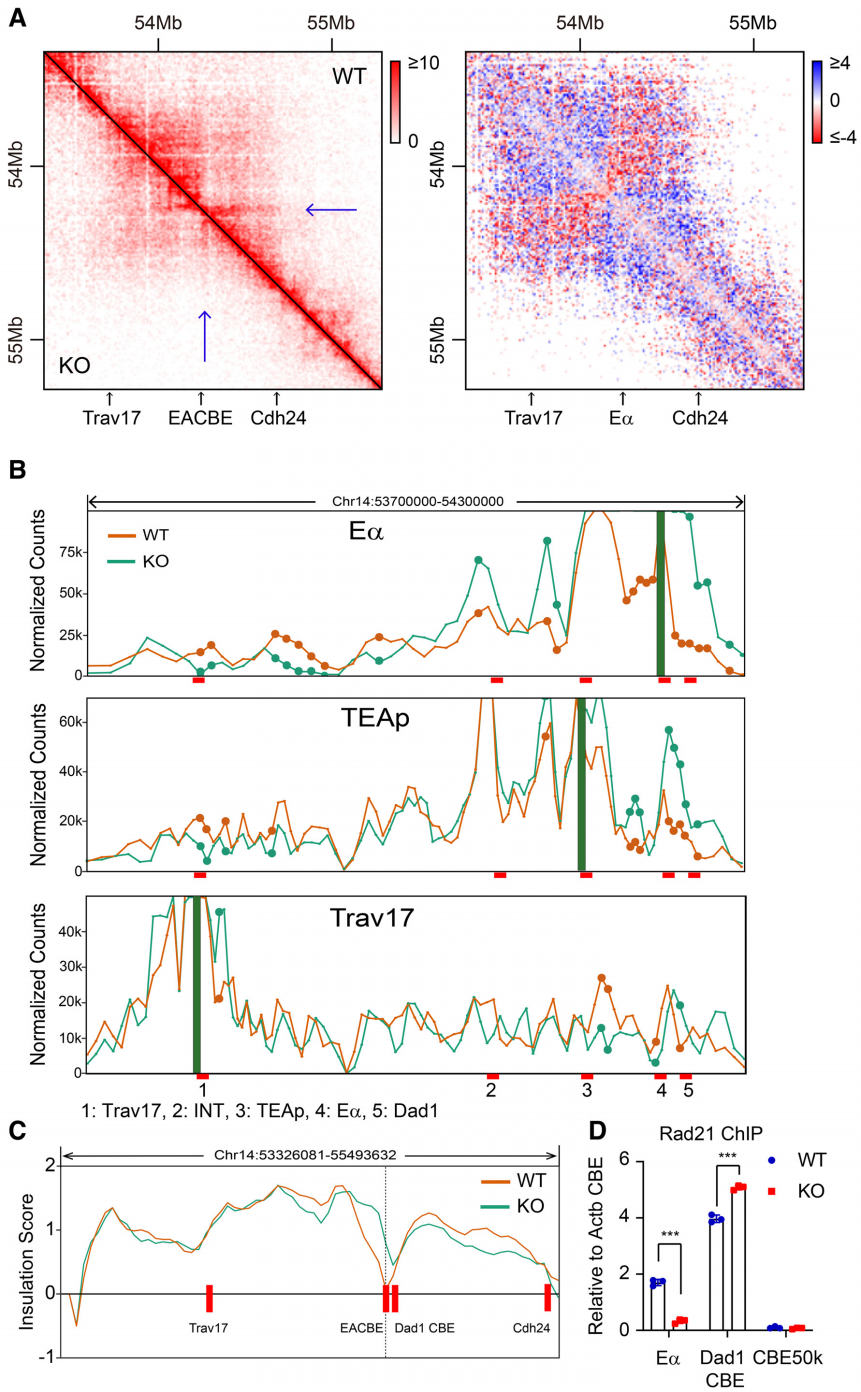
EACBE deletion impaired the interactions between the proximal Vα and the 5′ Jα genes. (**A**) Heatmap and subtraction heatmap of 10 kb binned Hi-C data of DP thymocytes generated from anti-CD3 injected EACBE^+/+^ × Rag1^−/-^ and EACBE^−/−^ × Rag1^−/-^ mice. (**B**) 4C signal normalized using 4C-ker program from Eα (EACBE left), TEAp and Trav17 viewpoint in CD3-stimulated-DP thymocytes of WT and EACBE^−/−^ mice at Rag2^−/−^ background. It was analyzed with two independent replicates. Filled circles highlight significant differences. (**C**) Insulation profiles of the *Tcra*-*Tcrd* locus. (**D**) Cohesin occupancy on the Eα was assessed by using ChIP-qPCR. Values of bound/input were normalized to the *Actb* promoter CBE. Data are mean ± SD of three PCR replicates of one of two independent experiments. *** *P*< 0.005 by two side Student's *t* test.

To further test whether EACBE deletion alone changes the chromatin structure of the *Tcra–Tcrd* locus, we performed a Hi-C experiment with DP thymocytes from EACBE^−/−^ × Rag1^−/−^ mice. The sub-TADs boundary disappeared, and the two sub-TADs merged in EACBE deficient DP thymocytes, while other regions kept intact (Figure 5A and Supplementary Figure S7b). The interactions within the sub-TADs are reduced while the interactions crossing the boundary increased, which suggested reduced the proximity of the proximal Vα gene region to the Jα array. To quantify the interaction change in EACBE-deleted thymocytes, we performed 4C assays and statistical analysis of interaction changes using the 4Cker program (32). We observed increased interactions of Eα with the *Dad1* and INT1–2, but the significantly reduced interactions with the proximal Vα gene region (Figure 5B). Interactions between TEA and the proximal Vα genes like Trav17 were reduced in both the TEA and Trav17 viewpoints (Figure 5B). This may explain the reduced accessibility of the proximal Vα genes.

The EACBE is located at the bottom of the valley between *Tcra* and *Dad1* genes on the basis of insulation scoring (Figure 5C). Insulation activity was reduced in EACBE-deleted thymocytes, and moved downstream to the CTCF binding site of the *Dad1* gene (Figure 5C). The 4C data confirmed the reduced insulation activity of EACBE-deleted allele. All baits located to the left of the EACBE had significantly increased interactions with the fragments to the right of the EACBE, including the *Dad1* CBE, on EACBE-deleted alleles (Figure 5B, Supplementary Figure S7c). We also observed increased interactions of the EACBE right fragment with the TEAp, INT1–2, and even some Vα genes on EACBE-deleted alleles (Supplementary Figure S7d). The cohesin occupancy on the *Dad1* CBE increased in the EACBE-deleted allele using ChIP-qPCR (Figure 5D), which suggested that more cohesin rings from the *Tcra-Tcrd* region crossed the boundary.

### The EACBE regulates expression of the genes at the downstream region

**Figure 6.**
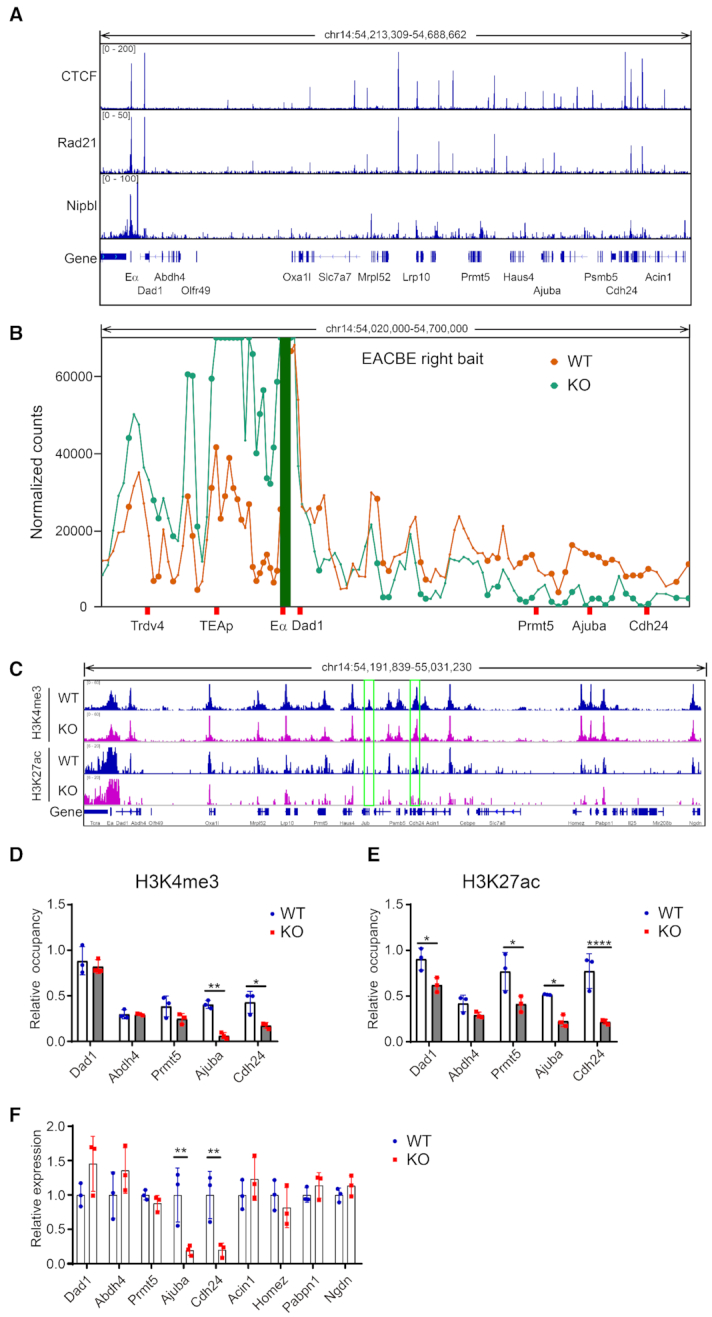
EACBE regulates the genes in the downstream region. (**A**) CTCF, Rad21 and Nipbl ChIP-seq signals are plotted for the downstream region (**B**) 4C signal normalized by 4C-ker program from Eα downstream viewpoints in DP thymocytes generated from anti-CD3 injected WT and EACBE^−/−^ mice at Rag2^−/−^ background. It was analyzed with two independent replicates. Filled circles highlight significant differences. (**C**) H3K4me3 and H3K27ac ChIP-seq signals. The ChIP-seq data are representative of two independent experiments. (**D**) and (**E**) ChIP-qPCR of H3K4me3 and H3K27ac on EACBE downstream region in DP thymocytes. The ChIP-qPCR data represent mean ± SD of three experiments, with normalization to values for the *Actb* promoter. (**F**) Relative transcription of the genes in the downstream region. The expressions are normalized to the *Actb* gene and then to WT. The data represent mean ± SD of three experiments for WT and EACBE^−/−^. * *P*< 0.05, ** *P*< 0.01, **** *P*< 0.001 by two side multiple Student's *t* test.

We noticed that the stripe from the Eα-Dad1 extended to the *Cdh24* gene at around 400kb downstream of the EACBE, and the strength and length of the stripe was reduced in EACBE-deleted thymocytes (Figure 1A and 5A). There are several CTCF binding sites at the *Cdh24* gene, where Hi-C data also showed a TAD boundary (Figure 5A). We also noticed that there is a strong Nipbl binding site at the right of the EACBE (Figure 6A). The Nibpl peak is located in the regulatory region of LCR HS3–6 (33). According to asymmetric extrusion theory, cohesin can be loaded at the Nipbl binding site and slide along toward to *Cdh24* gene, which may regulate the genes in the downstream TAD.

We tested interactions of the EACBE with the genes in the downstream TAD. Interactions between the EACBE right (Figure 6B), Dad1 CBE left, and EACBE left (Supplementary Figure S8) viewpoints and the *Prmt5*, *Ajuba* and *Cdh4* genes were reduced significantly in EACBE-deleted thymocytes. Nevertheless, the EACBE left (Eα) and EACBE right viewpoints showed increased interactions with the proximal region including the *Dad1* gene in EACBE-deleted cells (Supplementary Figure S8) (Figure 6B).

We noticed that the signals of the histone modification H3K4me3 and H3K27ac reduced at the *Ajuba* and *Cdh24* genes in EACBE-deleted thymocytes (Figure 6c). These reductions were confirmed by ChIP-qPCR (Figure 6D and E). To confirm a regulatory role of EACBE on the distal genes, we performed reverse-transcription qPCR. We observed increased expression of the *Dad1* gene and reduced expression of the *Ajuba* and *Cdh24* genes in EACBE-deleted thymocytes in a *Rag2*-deficient background (Figure 6F), consistent with the changes in interactions noted above. But the genes (*Acin1*, *Homez*, *Pabpn1* and *Ngdn*) in the downstream of the Cdh24 gene weren’t affected by the EACBE deletion (Figure 6F), indicating that the EACBE’s function was limited in the sub-TAD.

**Figure 7.**
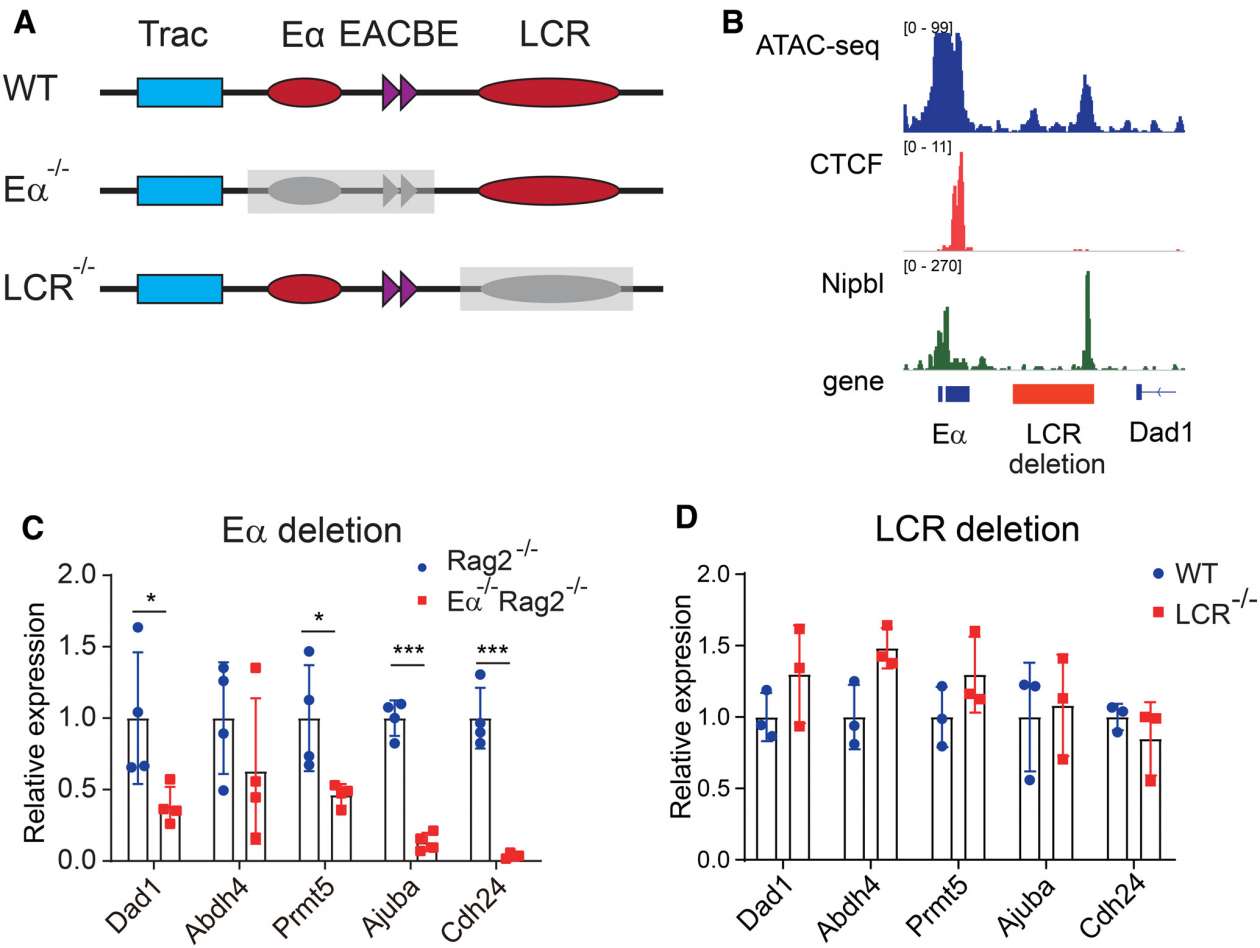
The Eα regulates expression of the genes at the downstream region. (**A**) Schematic of the Eα and LCR deletion. (**B**) The LCR deletion region includes two ATAC peaks, one of which is overlapped with Nibpl peak. Red line represents the LCR deletion region. (**C** and **D**) Relative transcription of the genes in the downstream region in DP thymocytes from Eα-deleted and LCR-deleted mice. The expressions are normalized to the *Actb* gene and then to WT. The data represent mean ± SD of four (Eα-deleted mice) or three (LCR-deleted mice) experiments. * *P*< 0.05, *** *P*< 0.005 by two side multiple Student's *t* test.

To determine the mechanism by which EACBE regulates gene expression in the downstream region, we also evaluated gene expression in Eα^−/−^ thymocytes on a *Rag2*-deficient background, noting again that this mutation deletes both Eα and EACBE (Figure 7A–C). The results showed that all genes in the downstream TAD were expressed at lower levels when Eα and EACBE are deleted as compared to either WT or EACBE-deleted thymocytes (Figure 7C). This suggests that in WT, Eα has a modest positive influence on gene expression across this region that is constrained by EACBE insulation for proximal genes (*Dad1* and *Prmt5*) but may be facilitated by EACBE for distal genes (*Ajuba* and *Cdh24*).

We also generated the LCR deleted allele, which deleted the region containing the two ATAC-seq peaks and the EACBE right Nipbl peak (Figure 7A, B). When we looked the expression of the downstream genes, we found that the LCR deletion did not change the expression of the genes (Figure 7D). The data suggest that Eα and EACBE, but not the LCR, regulate genes in the downstream TAD.

## DISCUSSION

The initiation of *Tcra* rearrangement requires establishment of the recombination center at the 5′ end of the Jα array and access of the proximal Vα genes to the recombination center (2,4). Previous studies showed that *cis*-elements TEA and Eα play critical roles in establishment of the recombination center (5,7). Eα was shown to be essential for Vα-Jα rearrangement and for interactions between the proximal Vα genes and the Jα array (7,9) and to regulate histone modifications and transcription of both Jα segments and proximal Vα gene segments (30,31). However, these conclusions all derive from analysis of an Eα-deleted allele that eliminated not only the core enhancer, but flanking CBEs as well. Here, we have addressed the specific contribution of the EACBE to locus regulation. We demonstrate EACBE plays an important role in organizing *Tcra* locus chromatin and collaborates with the Eα core to have specific effects on the Vα repertoire. Moreover, EACBE has complex effects on unrelated downstream genes, in some cases insulating and in other cases augmenting their activation by the core Eα.

Many studies have reported that there are CTCF binding sites between V gene array and (D)J gene segments of antigen receptor genes (34), such as IGCR of *IgH* (35), Sis/Cer of *Igκ* (36–39), C1/C2 of *Tcrb* (40) and INTs of *Tcrd* (8). These CTCF binding sites function as a barrier in restraining RAG scanning and rearrangement of the proximal V genes, normalizing recombination of D/J segments with all V segments to generate diverse repertoire (31). However, the Tcra rearrangement undergoes in a manner of stepwise proximal-to-distal progressions of Vα and Jα use in a single allele, which starts at the proximal Vα-to-Jα rearrangement and can undergo several rounds (28). The multiple rounds of V-J rearrangement increase the opportunity of TCRαβ thymocytes in positive selection. EACBE promotes the interaction and rearrangement of the proximal Vα and Jα segments through the establishment of the sub-TAD boundary, which is different from those CBEs located between V and D/J segments.

The EACBE is located at the sub-TADs boundary which separates the *Tcra–Tcrd* locus and the downstream region including *Dad1* gene. However, EACBE plays dual roles in the two sub-TADs: promoting the Eα interacting with the proximal Vα genes and the distal genes in the downstream sub-TAD. The two functions seem to be mutually exclusive, and Eα could not interact with the two regions at the same time. The theory of CTCF binding site orientation and loop extrusion may explain the role of the Eα on the downstream genes. But how EACBE regulates the dynamic chromatin organization of the *Tcra*-*Tcrd* locus is unknown, although the sub-TAD boundary seems to make the *Tcra–Tcrd* locus more compact. Several studies reported that cohesin, CTCF, or WAPL depletion or CTCF binding loss mainly affect the expression of the genes near TAD boundaries or CTCF binding sites (18,27,41–42). However, we found that the genes most affected are those near the other TAD boundary, not those near the boundary itself. Whether it is universal in genome needs further study.

The previous report showed that *Tcrd* rearrangement shapes *Tcra* repertoire (8,28). Most of *Tcrd* rearrangement events delete the INT1–2, which may abrupt the insulating activity of the INT1–2. The deletion also shortens the linear distance of Vα genes to Jα array. There are many CTCF binding sites in the Vα gene region and the chromatin extrusion from these CBE may promote the access of the Vα genes to the recombination centre at the Jα array (12). So, it is possible that the EACBE may also play a role of the primary rearrangement in the *Tcrd*-rearranged allele and the secondary rearrangement. We also observed a change of the Vδ usage in EACBE-deleted thymocytes. So, there are multiple factors involved in shaping the *Tcra* repertoire in EACBE-deleted thymocytes.

We saw an increased expression of *Dad1* gene in thymocytes of EACBE^−/−^ mice, which is consistent with the observation in the cohesin deficient DP thymocytes (43). The increased *Dad1* expression in EACBE-deleted DP thymocytes can be explained by the increased interaction between the enhancer Eα and the *Dad1* gene. We also noticed that EACBE is involved in the regulation of the distal genes like *Ajuba* and *Cdh24* at the far downstream of the EACBE. There are two Nipbl sites flanking the EACBE at the Eα and LCR respectively. We had speculated that the Eα act on the upstream *Tcra* region and the LCR on the downstream region. But the result showed that it is Eα but not LCR that regulates the downstream region.

Several genes in the downstream TAD such as *Dad1* and *Prmt5* are involved in the development and function of T lymphocytes. The Dad1 is an N-glycosylation regulatory protein and has been reported to play a role in preventing apoptotic cell death in embryogenesis (44,45). The *Dad1* gene is also expressed in thymocytes and its overexpression influenced T cell activation (46). The *Prmt5* gene encodes an arginine methyltransferase catalyzing arginine symmetric dimethylation of many proteins including histones H4 (at R3) and H3 (at R8) to NF-κB and spliceosome proteins (47,48). Prmt5 has been showed to promote proliferation and survival of cancer cells, stem cells, and lymphocytes (49–51). Deletion of *Prmt5* in T lymphocytes impaired development of thymocytes and peripheral T cells (52). The cell numbers of DP, SP and T_reg_ cells reduced in *Prmt5*-knockout mice, and even in the heterozygous mice (52). The reduced *Prmt5* expression in the Eα-deleted thymocytes suggested a possibility of that the Eα may regulate cell proliferation at least in DP thymocytes. The *Ajuba* gene encodes a versatile scaffold participating in several major signaling pathway to execute multiple physiological functions including cell adhesion, motility, mitosis, survival, and mechanical force sensing (53). We speculate that the Eα may do a fine regulation of cellular proliferation and survival by directly regulating the genes in the downstream sub-TAD except its role on *Tcra* rearrangement.

Hong NA *et al.* deleted the *Tcra* locus control region (LCR) HS2 to HS6 (NΔ26) including EACBE and four HS sites and observed lethality of homozygous mutant embryos due to the abnormal expression of the *Dad1* gene (45). But our LCR deletion mice develop normally and the expressions of the genes in the downstream region including *Dad1* are not affected in DP thymocytes. But we did see reduced expression of the *Dad1* gene in the Eα-deleted mice.

Here, we explored a role of the Eα CTCF binding site EACBE in the chromatin dynamic organization of the around 1Mb region from the proximal Vα region to the downstream *Cdh24* gene. The EACBE establishes a sub-TAD boundary which separates the *Tcra–Tcrd* locus and the downstream region. It helps the Eα core to regulate the select proximal Vα genes and normal *Tcra* rearrangement. It also makes the Eα able to regulate the far downstream genes *via* asymmetric chromatin extrusion. This study provides a new insight into the role of CTCF binding sites at TAD or sub-TAD boundaries in gene regulation.

## DATA AVAILABILITY

4C, RACE-seq, ChIP-seq and Hi-C data are available in GEO database as GSE145147. Rad21, Nipbl and CTCF ChIP-seq were downloaded from GEO database as GSE48763 and GSE41743.

## Supplementary Material

gkaa711_Supplemental_File
