# Supplementary material for: A role of the CTCF binding site at enhancer Eα in the dynamic chromatin organization of the Tcra–Tcrd locus
Source: Nucleic Acids Res. 2020 Aug 27;48(17):9621–36. doi: 10.1093/nar/gkaa711 (PMC7515734; doi:10.1093/nar/gkaa711)
Supplement: gkaa711_Supplemental_File [file gkaa711_supplemental_file.pdf]

Supplementary Materials:

Fig. S1 The EACBE insulates the interactions between the left and the right DNA fragments. Quantification of the interactions of 4C data from the left viewpoint and the right view point of EACBE was done with 4C-ker program.

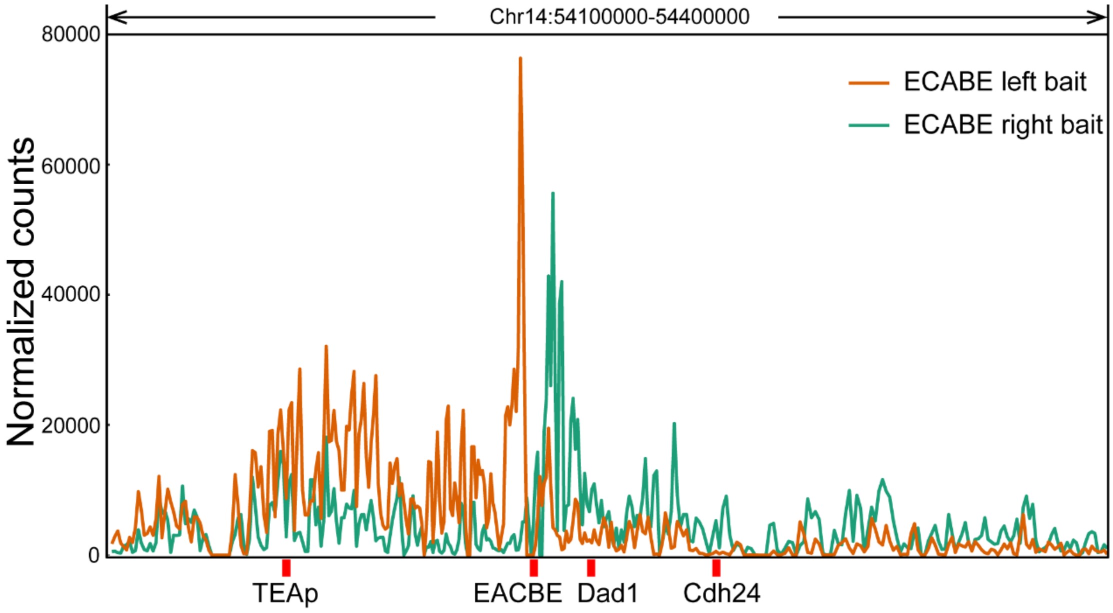

Fig. S2 The chromatin architecture stripes in non-T cells. A) Hi-C heatmap of mESC, NPC, and neuron cells with 10kb resolution of the region spanning from the proximal Vα region to around 300kb downstream of Cα. B) Hi-C heatmap of mESC-CTCF-auxin cells, in which CTCF was degraded, and control E14D0 cells. It was presented in Hi-C data browser (http://promoter.bx.psu.edu/hi-c/view.php). mESC, NPC and neuron: mouse embryonic stem cells, neural progenitors and cortical neurons from the developing mouse embryonic neocortex. E14D0: Mouse ES cell lines were derived from E14 strain. mESC-CTCF-auxin: CTCF depleted with auxin-inducible degron system for two days.

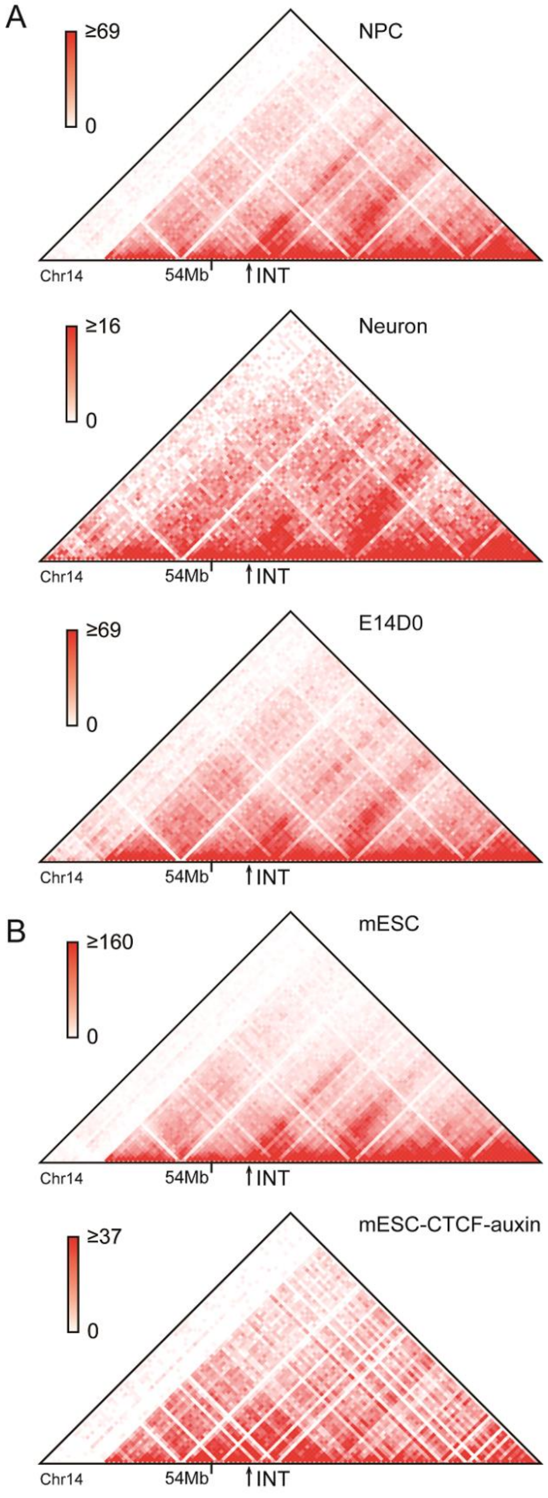

Fig. S3 EACBE deletion didn't influence with thymocyte development and TCR expression on surface. A) Percentages of thymocyte subsets and B) DN subsets from 6-week-old wild type and EACBE$^{-/-}$ mice were analyzed by using flow cytometry. Data represent mean ± SD of three independent experiments. C) Flow cytometry plot and D) Cell numbers of CD4^{+} and CD8^{+} lymphocytes in spleen of 6-week-old wild type and EACBE$^{-/-}$ mice were analyzed by using flow cytometry. The flow cytometry dot plots show gating of CD4^{+} and CD8^{+} cells in CD3^{+} population. E) Flow cytometry showed the TCRβ and CD3 on cell surface. Data are two independent experiments.

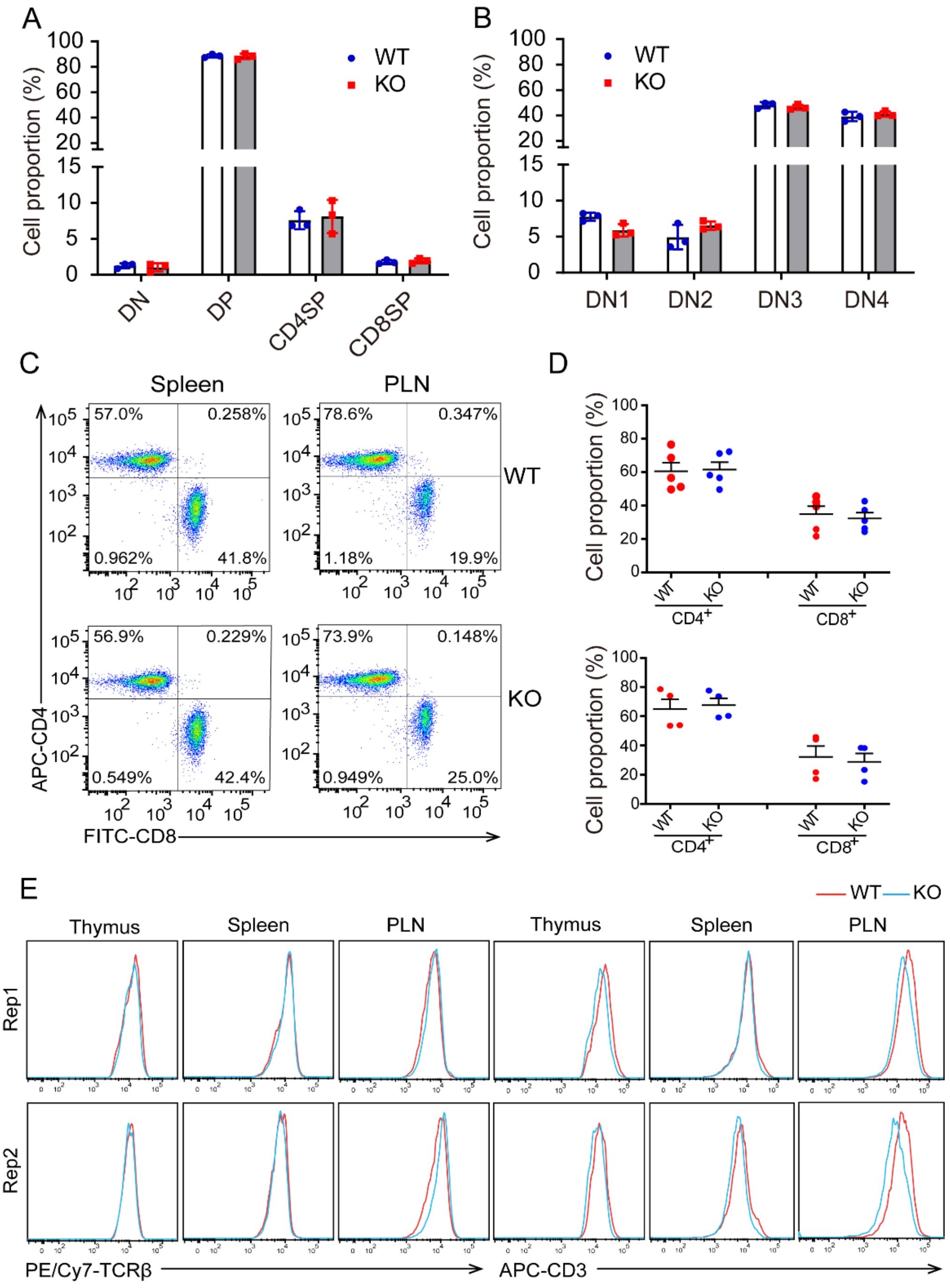

Fig. S4 Activation of EACBE-deleted T cells. A) Flow cytometry of CD4$^+$ and CD8$^+$ T cell from spleen or lymph node of wild type and EACBE$^{-/-}$ mice before and after 24-hour plate-bound CD3/CD28 stimulate. Cell proportion of activated CD4$^+$ and CD8$^+$ T cells from B) spleen and C) peripheral lymph node after 24-hour stimulate. Data are representative of seven (spleen) or four (PLN, peripheral lymph node) independent experiments (one mouse per experiment). * P < 0.05 by two-side Student's T test.

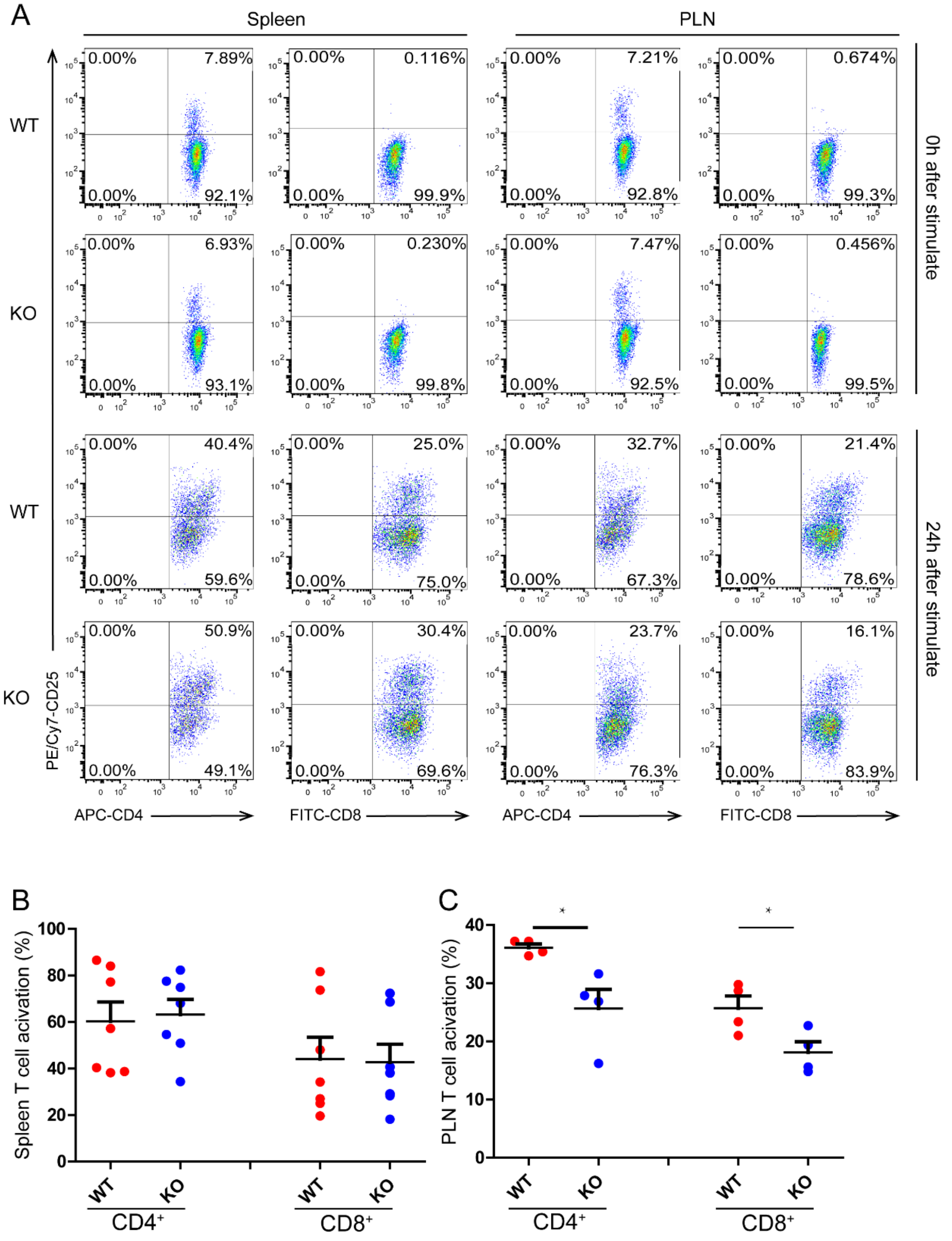

Fig. S5 The repertoire of *Tcrb* and *Tcrd* in EACBE$^{-/-}$ thymocytes. Relative clonotype numbers of A) Jβ, B) Vβ genes and C) heatmap of Vβ–Jβ rearrangements determined by deep sequencing of *Tcrb* transcripts amplified by 5'RACE of wild type and EACBE$^{-/-}$ mice respectively. Data are representative of two independent experiments. D) Vδ usage determined by high-throughput sequencing of *Tcrb* transcripts amplified by 5'RACE of wild type and EACBE$^{-/-}$ mice respectively. Data are mean ± SD of two experiments. * $P < 0.05$, ** $P < 0.01$ by two side Student's T test.

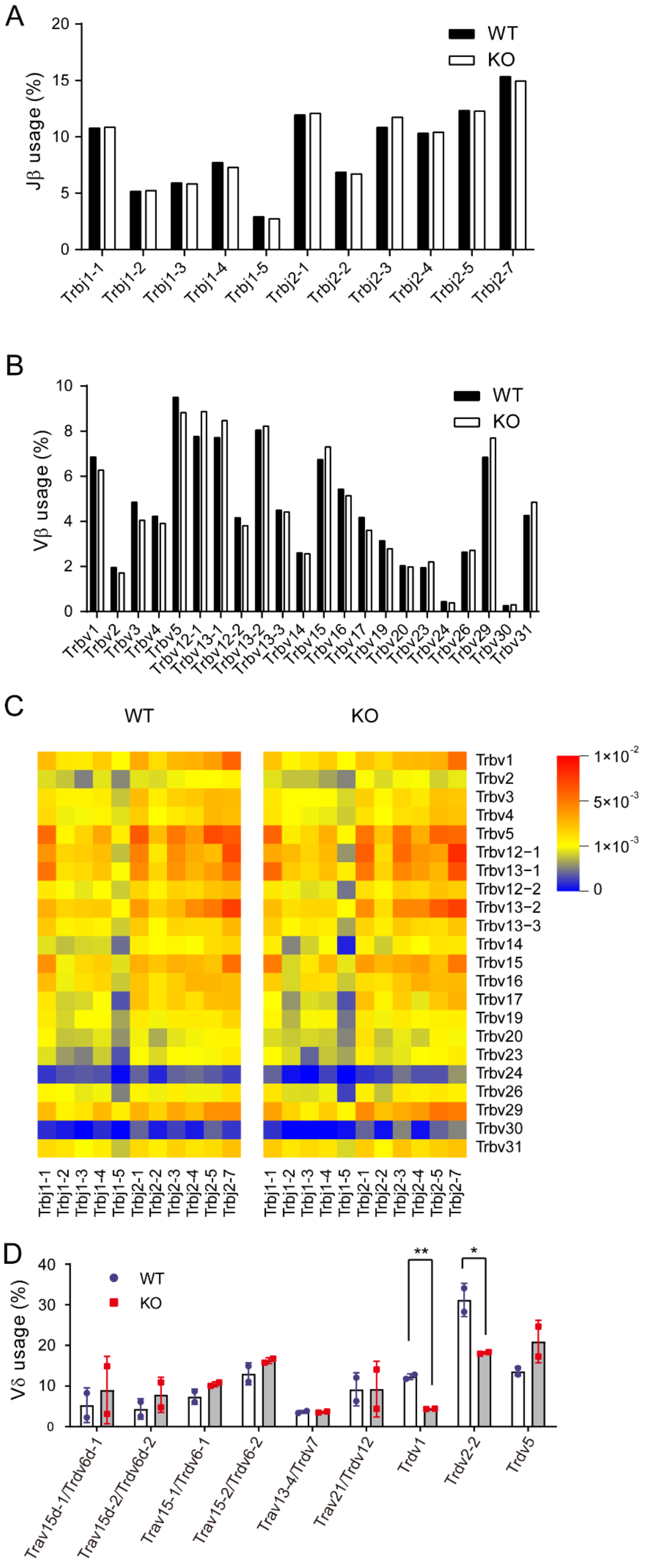
A
Jβ usage (%)
WT
KO
0
5
10
15
20
Trbj1-1
Trbj1-2
Trbj1-3
Trbj1-4
Trbj1-5
Trbj2-1
Trbj2-2
Trbj2-3
Trbj2-4
Trbj2-5
Trbj2-7
B
Vβ usage (%)
WT
KO
0
2
4
6
8
10
Trbv1
Trbv2
Trbv3
Trbv4
Trbv5
Trbv12-1
Trbv13-1
Trbv12-2
Trbv13-2
Trbv13-3
Trbv14
Trbv15
Trbv16
Trbv17
Trbv19
Trbv20
Trbv23
Trbv24
Trbv26
Trbv29
Trbv30
Trbv31
C
WT
KO
1×10^-2
5×10^-3
1×10^-3
0
Trbv1
Trbv2
Trbv3
Trbv4
Trbv5
Trbv12−1
Trbv13−1
Trbv12−2
Trbv13−2
Trbv13−3
Trbv14
Trbv15
Trbv16
Trbv17
Trbv19
Trbv20
Trbv23
Trbv24
Trbv26
Trbv29
Trbv30
Trbv31
Trbj1-1
Trbj1-2
Trbj1-3
Trbj1-4
Trbj1-5
Trbj2-1
Trbj2-2
Trbj2-3
Trbj2-4
Trbj2-5
Trbj2-7
D
Vδ usage (%)
WT
KO
0
10
20
30
40
**
*
Trav15d-1/Trdv6d-1
Trav15d-2/Trdv6d-2
Trav15-1/Trdv6-1
Trav15-2/Trdv6-2
Trav13-4/Trdv7
Trav21/Trdv12
Trdv1
Trdv2-2
Trdv5

Fig. S6 EACBE deletion didn't influence thymocyte survival. A) Flow cytometry plot of apoptosis assay of thymocytes cultured on 0, 6 hours, 24 hours, and 48 hours in medium with 10% FBS. Data are representative of three independent experiments. B) Apoptosis cell percentage and C) survival cell percentage of thymocytes after 0-hour, 6-hour, 24-hour-s, and 48-hour cultures. The data represent mean of three experiments with normalization to the 0-hour.

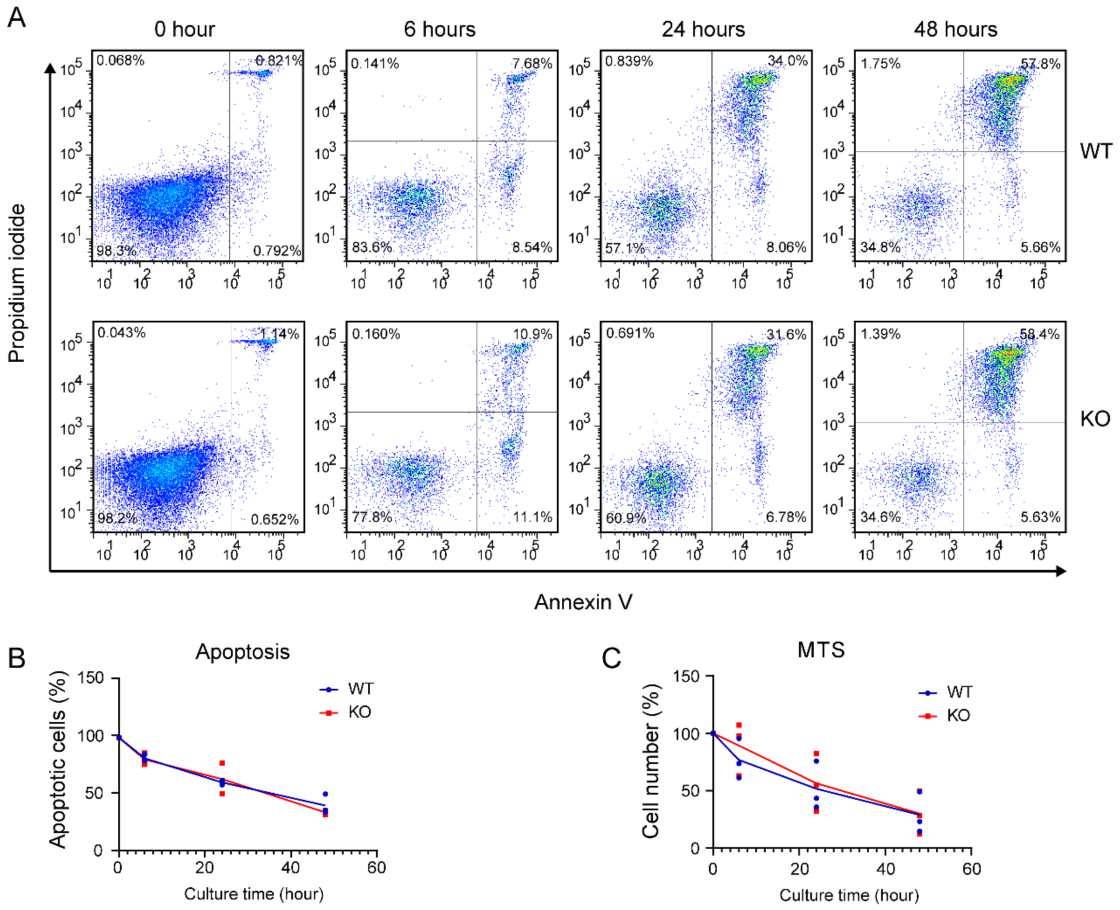

Fig. S7 EACBE is involved in chromatin organization of the *Tcra-Tcrd* locus. A) H3K4me3 and H3K27ac ChIP-seq on the *Actb* gene locus in Rag2$^{-/-}$ (WT) and Rag2$^{-/-}$ ×EACBE$^{-/-}$ (KO) DP thymocytes from anti-CD3 injected mice. Data are representative of two independent experiments. B) Heatmap and subtraction heatmap of 4Mb region on Chromosome 14. The 10 kb binned Hi-C data of DP thymocytes were generated from anti-CD3 injected EACBE$^{+/+}$×Rag1$^{-/-}$ and EACBE$^{-/-}$×Rag1$^{-/-}$ mice. 4C data normalized using 4C-ker program from C) EACBE right and D) INT viewpoint in CD3-stimulated-DP thymocytes of WT and EACBE$^{-/-}$ mice at Rag2$^{-/-}$ background. It was analyzed with two independent replicates. Filled circles highlight significant differences.

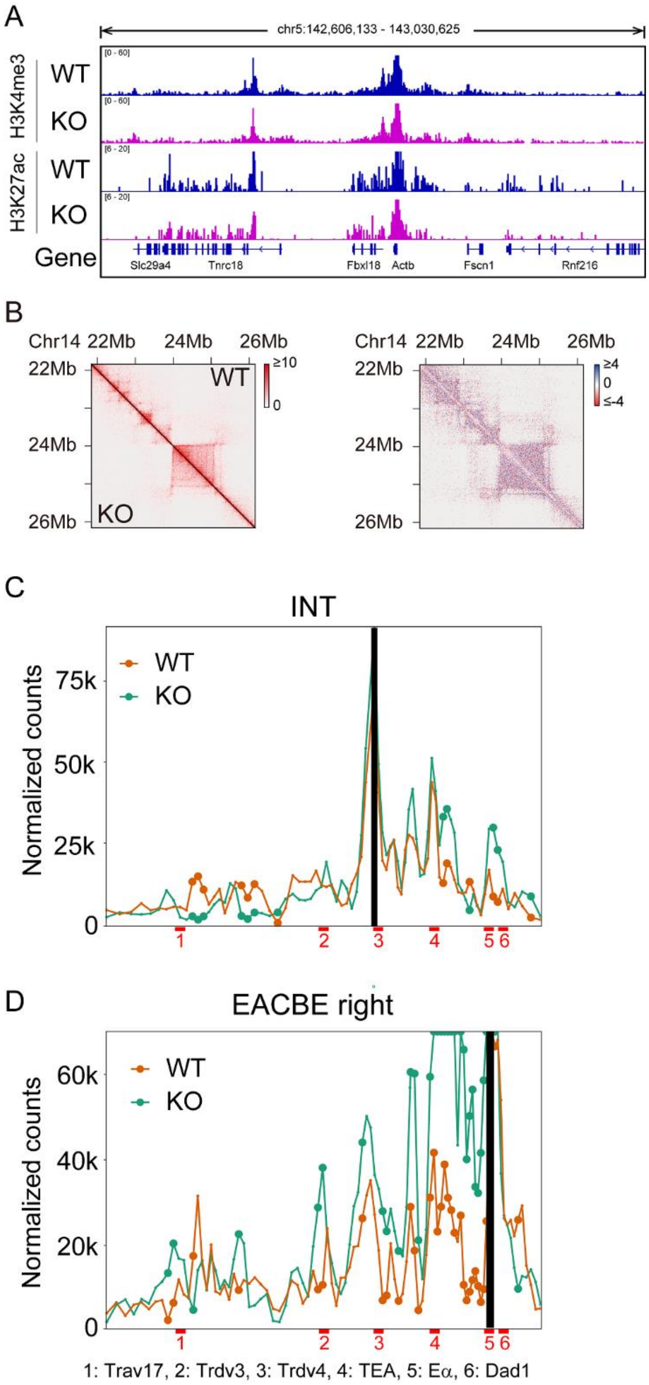

Fig. S8 EACBE mediates interactions of the Eα with the genes in the downstream sub-TAD. 4C data normalized using 4C-ker program from EACBE right and INT viewpoint in CD3-stimulated-DP thymocytes of WT and EACBE$^{-/-}$ mice at Rag2$^{-/-}$ background. It was analyzed with two independent replicates. Filled circles highlight significant differences.

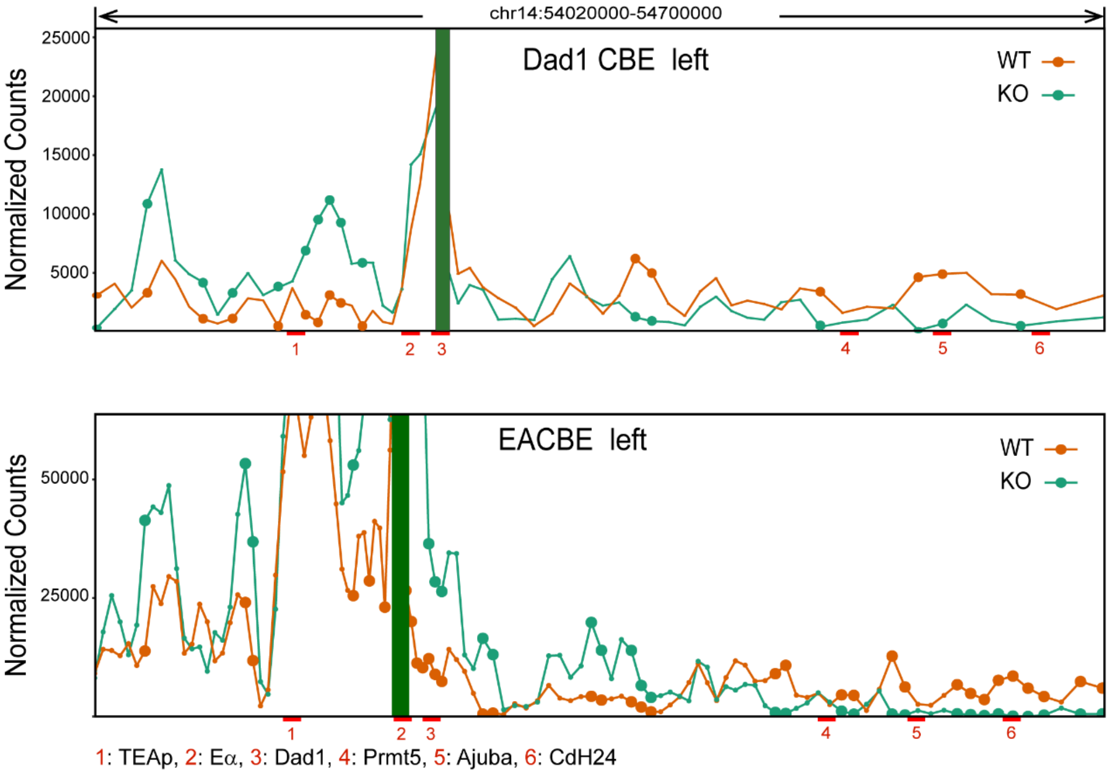

Table S1 the primer sequences used in the paper.

Primers	Sequence	Use/figure
		ChIP
musActbp F	5’-GCTGTGGCGTCCTATAAAACC	F2B,C;F4B,C;F5D
musActbp R	5’-CAACGAAGGAGCTGCAAAGAA	F2B,C;F4B,C;F5D
MageA2C F	5’-AACGTTTTGTGAACGTCCTGAG	F2C, F5B,C
MageA2B R	5’-GACGCTCCAGAACAAAATGGC	F2C, F5B,C
mus Ea F	5’-CTGACATGGGCAAACAGGTC	F2C; F4B,C
mus Ea R	5’-GTGGCCCGGAGAGATCTTAT	F2C; F4B,C
Ea50k/CBE50k F	5’-AGGACTTGGCACAACCTCTG	F2B; F5D
Ea50k/CBE50k R	5’-GCTCTCCCTGAATCTGTTGC	F2B; F5D
ChIP TEAp F	5’-ATGGGAAAGGGACCGATAAG	F4B,C
ChIP TEAp R	5’-GCTCAAAGGACACTGGAAGG	F4B,C
ChIP Trav17-F	5’-TCCCAGTGACCATTCTGCTG	F4B,C
ChIP Trav17-R	5’-TGTCCCTTGTGTAGATTCTGAC	F4B,C
ChIP Trav21-F	5’-TGTGGGGTTGCTGCTTGAAG	F4B,C
ChIP Trav21-R	5’-AACACTTACCCAAAGCCAGGAG	F4B,C
ChIP Trdv2-2F	5’-TCCTGTTTTGAAGGTGAGACAG	F4B,C
ChIP Trdv2-2R	5’-GCCTTCTTACCAAGAGAAGTGG	F4B,C
ChIP Trdd1 F	5’-TACGGCTGTGTTTCACTGTGAT	F4B,C
ChIP Trdd1 R	5’-GCTCAATGGACTCTTTGCAGTG	F4B,C
ChIP Trdj1 F	5’-AGCTGCTGAGGTTTTTGGAATG	F4B,C
ChIP Trdj1 R	5’-ATCCCTCAGACCCTAACCCAGA	F4B,C
ChIP Trdj2 F	5’-GCTGGTCCCAGACTGGTTATCT	F4B,C
ChIP Trdj2 R	5’-AACTTACGGGGCTCCACAAAG	F4B,C
ChIP Trdv5 F	5’-CTGGACTCTCTTTAACCATCCC	F4B,C
ChIP Trdv5 R	5’-TCCTTGGTAGAGGTATTCACCC	F4B,C
ChIP Traj61 F	5’-GCCATGACTGGAAGACTCAT	F4B,C
ChIP Traj61 R	5’-TCCATATTTTGTTACTATTCCTGAAG	F4B,C
ChIP Traj58 F	5’-TGGGTCTAAGCTGTCATTTGG	F4B,C
ChIP Traj58 R	5’-TTGACTGGACTTGAATTTTGG	F4B,C
ChIP Traj47 F	5’-GCTTGGGAACCATTTTGAGA	F4B,C
ChIP Traj47 R	5’-CACCTGGAGCTTTGTTTGTTT	F4B,C
ChIP Traj18 F	5’-AGAGCGGACAGAAGATCGTT	F4B,C
ChIP Traj18 R	5’-TATCTACACAGTGCCAGCCC	F4B,C
ChIP Traj7 F	5’-GTCCTCATAGCACCCCTTACA	F4B,C
ChIP Traj7 R	5’-AAAACGCACACATTTCGGCT	F4B,C
ChIP Traj2 F	5’-TCTAAGGAGTAGGTAGATCGCC	F4B,C
ChIP Traj2 R	5’-GGTCCCTTCCCCGAATGTTA	F4B,C
Ea 3’ F	5’-TTCCAGCGGGATACCTGTTA	F2B; F5D
Ea 3’ R	5’-ACCCCTTTGGCCATTTCTTA	F2B; F5D
Dad1 CBE F	5’-CAGCACAGGTGAGGAAGACA	F5D
Dad1 CBE R	5’-GACCAGGGGTTTCTTTCCAT	F5D
ACTB CTCF F	5’-ACGATGGAGGGGAATACAGC	F5D; F6D; F6E

ACTB CTCF R	5’-TGATAGTTCGCCATGGATGAC	F5D; F6D; F6E
Dad1_p F	5’-GAGCTCAAGTACTCCTCCAGG	F6D; F6E
Dad1_p R	5’-GTATCCGAAGTCCCCGTGTT	F6D; F6E
Abdh4_p F	5’-TCAAGTTCCTCCGCAGTGAG	F6D; F6E
Abdh4_p R	5’-CCCAGATTCAGCCTTCCAGT	F6D; F6E
Prmt5_p F	5’-GCCCAGAGATCCAACCAACT	F6D; F6E
Prmt5_p R	5’-GTGAAAGTGATGTGCCCCAA	F6D; F6E
Ajuba_p F	5’-GGATTTCCTGGGCATCACA	F6D; F6E
Ajuba_p R	5’-CAATCTGCCCCTTGACCTTT	F6D; F6E
Cdh24_p F	5’-AGGGCAAGAGGGAGAGAGAA	F6D; F6E
Cdh24_p R	5’-TCCCATCTCACACACCTCTC	F6D; F6E
		DNA Jα usage
Trav12F	5’-GCAGCAGCTCCTTCCATC	F3D
Trav13F	5’-AAGAACGTCGCAGCTCTTTG	F3D
Trav14F	5’-TGGAGACTCAGCCACCTACT	F3D
Trav17F	5’-CCAGCCCATTGGAGCGACT	F3F
Trav19F	5’-CATCACAGCCTCCCAGCCT	F3F
Trav21F	5’-CACCTTGATCCTGCCTCATGT	F3F
Traj61R	5’-ATGAGTCTTCCAGTCATGGC	F3D,F
Traj57R	5’-AGCTCACTGTCAGCTTTGTCC	F3D,F
Traj53R	5’-GGAGTCACAGTTAAGAGAGTTCC	F3D
Traj49R	5’-GGAATGACAGTCAAACTTGTTCC	F3D
Traj40R	5’-TGGTACCTGCTCCAAAGACG	F3D
Traj37R	5’-AAATGAGCATAAGCGACAG	F3D
Traj31R	5’-GCGTCCCATCACCAAAGAAG	F3D
Traj17R	5’-TGATGGCTAGGCTCCTTTTC	F3D
Traj2R	5’-TACCGGGTTGCAAATGGTG	F3D
Actb-pF	5’-CGCCATGGATGACGATATCG	F3D
Actb-pR	5’-CGAAGCCGGCTTTGCACATG	F3D
		5’RACE
5’ PCR IIA A501	5’-TGAACCTTAAGCAGTGGTATCAACGCAGAG	F3A,B
5’ PCR IIA A502	5’-TGCTAAGTAAGCAGTGGTATCAACGCAGAG	F3A,B
5’ PCR IIA A503	5’-TGTTCTCTAAGCAGTGGTATCAACGCAGAG	F3A,B
5’ PCR IIA A504	5’-TAAGACACAAGCAGTGGTATCAACGCAGAG	F3A,B
5’ PCR IIA A505	5’-CTAATCGAAAGCAGTGGTATCAACGCAGAG	F3A,B
5’ PCR IIA A506	5’-CTAGAACAAAGCAGTGGTATCAACGCAGAG	F3A,B
5’ PCR IIA A507	5’-TAAGTTCCAAGCAGTGGTATCAACGCAGAG	F3A,B
5’ PCR IIA A508	5’-TAGACCTAAAGCAGTGGTATCAACGCAGAG	F3A,B
TRAC-R（N701)	5’-TAAGGCGAACACAGCAGGTTCTGGGTTC	F3A,B
TRBC-R（N701)	5’-TAAGGCGAGGTGGAGTCACATTTCTCAG	S3A,B
TRDC-R（N701)	5’-TAAGGCGAGAAAACAGATGGTTTGGCCG	S5D
TRAC-R（N702)	5’-CGTACTAGACACAGCAGGTTCTGGGTTC	F3A,B
TRBC-R（N702)	5’-CGTACTAGGGTGGAGTCACATTTCTCAG	S5A,B
TRDC-R（N702)	5’-CGTACTAGGAAAACAGATGGTTTGGCCG	S5D

TRAC-R（N703)	5’-AGGCAGAAACACAGCAGGTTCTGGGTTC	F3A,B
TRBC-R（N703)	5’-AGGCAGAAGGTGGAGTCACATTTCTCAG	S5A,B
TRDC-R（N703)	5’-AGGCAGAAGAAAACAGATGGTTTGGCCG	S5D
TRAC-R（N704)	5’-TCCTGAGCACACAGCAGGTTCTGGGTTC	F3A,B
TRBC-R（N704)	5’-TCCTGAGCGGTGGAGTCACATTTCTCAG	S5A,B
TRDC-R（N704)	5’-TCCTGAGCGAAAACAGATGGTTTGGCCG	S5D
TRAC-R（N705)	5’-GGACTCCTACACAGCAGGTTCTGGGTTC	F3A,B
TRBC-R（N705)	5’-GGACTCCTGGTGGAGTCACATTTCTCAG	S5A,B
TRDC-R（N705)	5’-GGACTCCTGAAAACAGATGGTTTGGCCG	S5D
TRAC-R（N706)	5’-TAGGCATGACACAGCAGGTTCTGGGTTC	F3A,B
TRBC-R（N706)	5’-TAGGCATGGGTGGAGTCACATTTCTCAG	S5A,B
TRDC-R（N706)	5’-TAGGCATGGAAAACAGATGGTTTGGCCG	S5D
TRAC-R（N707)	5’-CTCTCTACACACAGCAGGTTCTGGGTTC	F3A,B
TRBC-R（N707)	5’-CTCTCTACGGTGGAGTCACATTTCTCAG	S5A,B
TRDC-R（N707)	5’-CTCTCTACGAAAACAGATGGTTTGGCCG	S5D
TRAC-R（N710)	5’-CGAGGCTGACACAGCAGGTTCTGGGTTC	F3A,B
TRBC-R（N710)	5’-CGAGGCTGGGTGGAGTCACATTTCTCAG	S5A,B
TRDC-R（N710)	5’-CGAGGCTGGAAAACAGATGGTTTGGCCG	S5D
		DSB
DSB linker1	5’-GCGGTGACCCGGGAGATCTGAATTC	F3E
DSB linker2	5’-GAATTCAGATC	F3E
Linker primer	5’-CCGGGAGATCTGAATTCCAC	F3E
DSB Traj61 primer	5’-CTGGAGAGAGAGGAGTGCTG	F3E
DSB Traj61 probe	5’-TGAGGAACACGGAGTATCTC	F3E
DSB Traj27 primer	5’-ATGGCAGATAGAATGGAGCGG	F3E
DSB Traj27 probe	5’-TACCTCCACCTGTCTTCTCA	F3E
DSB Traj18 primer	5’-CTGGCGGTGGAAAGACTATTG	F3E
DSB Traj18 probe	5’-TAAGCTCAGAGCGGACAGAA	F3E
DSB Traj6 probe	5’-GACCAATGGCAAAGGGAGGT	F3E
DSB Traj6 primer	5’-ATCAGACCAGACTGTCTGCCC	F3E
DSB Traj2 primer	5’-GTGGAGCCTCACAGTAGACCAG	F3E
DSB Traj2 probe	5’-CTCCACCCTGAGAGACTTTC	F3E
Cd14 F	5’-GCTCAAACTTTCAGAATCTACCGAC	F3E
Cd14 R	5’-AGTCAGTTCGTGGAGGCCGGAAATC	F3E
	Germline transcription	
GT-Trav17 F	5’-TGGAGCGACTCAGCCAAGTA	F4D
GT-Trav17 R	5’-CGTGCACAGAAGGTCTCAGG	F4D
GT-Trav19 F	5’-CCAGCCTGAAGACACAGCAG	F4D
GT-Trav19 R	5’-AGGGTCGCAGATGTCCTTGT	F4D
GT-Trav21 F	5’-CGGCTGTGTACCACTGTATCCT	F4D
GT-Trav21 R	5’-CTTGAAATGGATGCCTCTGCT	F4D
GT-Trdj1 F	5’-AGCTGCTGAGGTTTTTGGAATG	F4D
GT-Trdj1 R	5’-ATCCCTCAGACCCTAACCCAGA	F4D
GT-Trdv5 R	5’-ACCGACTGGAAGGATGATTCTT	F4D

GT-TEA F	5’-GCACCTTCCTTCCAAGATTCCT	F4D
GT-TEA R	5’-GTGCTGGTCCTTGTCTTTCGTT	F4D
GT-Traj56 F	5’-CCCTTGGAACCCTGATATGC	F4D
GT-Traj56 R	5’-CAGCCATTGTTTGGATTGGA	F4D
GT-Traj31 F	5’-GCAGCCTGCCAGCTATCTTT	F4D
GT-Traj31 R	5’-CAAAAGCAGCAACCCAACAA	F4D
GT-Traj23 F	5’-AGAGGAGGCCGAAAGTCTCC	F4D
GT-Traj23 R	5’-TGCACAAATCCAGGCCTATG	F4D
GT-Traj3 F	5’-TCTGGACCCTTGGCAATCAT	F4D
GT-Traj3 R	5’-CCCTGCCCTGGTCTACTGTG	F4D
GT-Trac F	5’-TGATGCCACGTTGACGTTTGAGA	F4D
GT-Trac R	5’-TCCATAGCTTTCATGTCCAGC	F4D
		Gene expression
Actb mRNA F	5’-ACACCCGCCACCAGTTC	F6F;F7C,D
Actb mRNA R	5’-TACAGCCCGGGGAGCAT	F6F;F7C,D
Dad1 mRNA F	5’-TGTGGGCAGCTTCATCCTAG	F6F;F7C,D
Dad1 mRNA R	5’-GTGCTGGCAAAGAGGAAGTC	F6F;F7C,D
Abdh4 mRNA F	5’-TGGAAGCCAGGATCCTCCAG	F6F;F7C,D
Abdh4 mRNA R	5’-CATCACCAGAGGGGTGCGAT	F6F;F7C,D
Prmt5 mRNA R	5’-GGTGGTTGGTTCCCGTGATG	F6F;F7C,D
Prmt5 mRNA F	5’-GCCATTCTCCCCACCAGCAT	F6F;F7C,D
Ajuba mRNA F	5’-TGCTCTGCCCATAGATACCT	F6F;F7C,D
Ajuba mRNA R	5’-GTCTCCTGGTCCCTTCGTTC	F6F;F7C,D
Cdh24 mRNA R	5’-CGTCTTCGGGCTCAATGGA	F6F;F7C,D
Cdh24 mRNA F	5’-GGGCCAGATCTCAGCCAGT	F6F;F7C,D
Acin1 mRNA F	5’-GATGGAGCTGCAGCCTCCT	F6F;F7C,D
Acin1 mRNA R	5’-CCCGTTCCGCGTCAAGCAG	F6F;F7C,D
Homez mRNA F	5’-AGCAGGTGCTCATTTCCATCC	F6F;F7C,D
Homez mRNA R	5’-AGCAGTCTCAACAGCTCTGCA	F6F;F7C,D
Pabpn1 mRNA F	5’-TCAAAGCTCGAGTCAGGGAGA	F6F
Pabpn1 mRNA R	5’-ACGTAGATAGAGCGGGCATCA	F6F
Ngdn mRNA F	5’-CACTGACGACAAAAGTTCGAGC	F6F
Ngdn mRNA R	5’-AGAGGCCTTGTCCAGGATGA	F6F
		4C primer
Ea-MboI2 up:	5’-TGGCGATGAAGTTGACTTTGATC	F1B;F5B;S1
Ea-NlaIII2 up:	5’-CAGGCAGAGACTCTTCGACG	F1B;F5B;S1
Ea-MboI1 down	5’-TGCCCATCATCCAGGTTCAGATC	F1B;F6B;S1
Ea-NlaIII1 down	5’-CTGGGTTTGCTGCACCTCAGT	F1B;F6B;S1
4C TEAp MboI:	5’-ACACTCTCTTTTCACAGCTGATC	F5B
4C TEAp NlaIII:	5’-GCGTTCTGATTTCCTTCACTTTG	F5B
4C TARV17 Mbo1	5’-CATTCCCTGGGATGGCAGATC	F5B
4C TARV17 Nla3:	5’-GTAATGGAAGGAGAGAGAAGGGT	F5B
4C INT MboI:	5’-GATCTACACAAAGCACTGCTGAAT	S7C
4C INT NlaIII:	5’-GGTTTCTGTGGTTGGAGTAGACT	S7C

4C EACBE right Mbo1 up	5’-GCCTGGGTCCTGCTAAGGATC	S7D
4C EACBE right Nla3 up:	5’-GGCAGAGAGCTAGACAGATGTAGT	S7D
